# SIOOT^®^ Adjunct Oxygen-Ozone Therapy Against Multidrug-Resistant Bacteria: A Pilot Study of 257 Cases

**DOI:** 10.3390/antibiotics15080768

**Published:** 2026-08-10

**Authors:** Marianno Franzini, Salvatore Chirumbolo, Giovanni Ricevuti, Luigi Valdenassi

**Affiliations:** 1 Italian Scientific Society of Oxygen-Ozone Therapy (SIOOT), 24020 Bergamo, Italy; marianno.franzini@gmail.com (M.F.); luigi.valdenassi@unipv.it (L.V.); 2 Department of Engineering for Innovation Medicine, University of Verona, 37134 Verona, Italy; 3 Department of Drug Science, University of Pavia, 27100 Pavia, Italy; giovanni.ricevuti@unipv.it

**Keywords:** ozone therapy, antibiotic resistance, therapy, SIOOT

## Abstract

**Background/Objectives:** Antimicrobial resistance (AMR) represents one of the greatest challenges to modern medicine, particularly in chronic infections sustained by multidrug-resistant (MDR) pathogens and biofilm formation. SIOOT^®^ Oxygen–ozone major autohemotherapy (SIOOT^®^-O_2_-O_3_-MAHT) has been proposed as an adjunctive treatment capable of exerting direct antimicrobial, antibiofilm, and immunomodulatory effects. This pilot study evaluated the clinical, microbiological, inflammatory, and mechanistic effects of standardized SIOOT^®^-O_2_-O_3_-MAHT administered alongside conventional antibiotic therapy in patients with chronic MDR bacterial infections. **Methods:** A prospective longitudinal pilot study was conducted in 257 patients with chronic infectious and inflammatory disorders refractory to prolonged antibiotic treatment. Patients received standardized SIOOT^®^-O_2_-O_3_-MAHT according to protocols from the Italian Scientific Society of Oxygen-Ozone Therapy (SIOOT) in addition to guideline-directed antibiotics. Longitudinal bacterial burden (CFU/mL), culture positivity, erythrocyte sedimentation rate (ESR), and C-reactive protein (CRP) were all assessed over a 12-month follow-up. In parallel, macrophage phagocytosis, intracellular bacterial killing, phago-lysosomal maturation, and methicillin-resistant *Staphylococcus aureus* (MRSA) biofilm disruption were investigated using confocal laser scanning microscopy, gentamicin protection assays, and scanning electron microscopy. **Results:** SIOOT^®^-O_2_-O_3_-MAHT was associated with a progressive reduction in bacterial burden from a geometric mean of 6.43 × 10^6^ CFU/mL before treatment to complete microbiological clearance after one year. Mean bacterial reduction reached 98.53% after one week and 99.95% after one month, while culture positivity decreased from 100% to 0% by one year (all *p* < 0.0001). ESR normalization increased from 28.2% at one week to 98.1% at one year, and CRP normalization increased from 32.4% to 98.7%. Mechanistic analyses demonstrated significantly enhanced macrophage phagocytosis, phago-lysosomal maturation, intracellular MRSA killing, and marked disruption of mature MRSA biofilms following ozone treatment. **Conclusions:** Adjunctive SIOOT^®^-O_2_-O_3_-MAHT was associated with reductions in bacterial burden, progressive improvement of systemic inflammatory markers, enhanced macrophage antimicrobial activity, and disruption of bacterial biofilms in patients with chronic multidrug-resistant infections. Given the prospective, non-randomized pilot design, these findings should be considered exploratory and hypothesis-generating, providing biological and clinical rationale for further investigation. Adequately powered randomized controlled trials are required to determine the efficacy, safety, and long-term clinical benefits of adjunctive oxygen–ozone therapy.

## 1. Introduction

Antimicrobial resistance (AMR) has become one of the defining biomedical threats of the twenty-first century. The most recent global estimates indicate that bacterial AMR was associated with 4.71 million deaths in 2021, including 1.14 million deaths directly attributable to resistant infections, with a projected rise to 1.91 million attributable deaths and 8.22 million associated deaths annually by 2050 if effective countermeasures are not implemented [1,2,3,4,5]. The World Health Organization 2024 Bacterial Priority Pathogens List further emphasizes the clinical urgency of Gram-negative bacteria resistant to last-resort antibiotics, together with drug-resistant *Mycobacterium tuberculosis*, *Salmonella*, *Shigella*, *Neisseria gonorrhoeae*, *Pseudomonas aeruginosa*, and *Staphylococcus aureus* [6]. Within this landscape, methicillin-resistant *S. aureus* (MRSA), vancomycin-resistant enterococci, extended-spectrum β-lactamase-producing Enterobacterales, carbapenem-resistant *Klebsiella pneumoniae*, multidrug-resistant *P. aeruginosa*, and carbapenem-resistant *Acinetobacter baumannii* remain particularly problematic because they combine genetic resistance with biofilm formation, persistence, metabolic plasticity, and immune evasion [7,8].

The failure of antibiotic therapy in Multi-Drug Resistant (MDR) infections is not merely a pharmacological problem. Although target modification, enzymatic degradation, efflux pumps, and reduced permeability remain central mechanisms of resistance, many refractory infections persist because bacteria occupy protected ecological niches within biofilms, hypoxic tissues, necrotic wounds, implanted devices, or poorly vascularized bone [9,10,11,12,13]. Biofilm-associated bacteria show reduced growth rates, altered metabolism, increased horizontal gene transfer, extracellular polymeric matrix protection, and enrichment of persister cells, all of which can substantially reduce antibiotic susceptibility despite apparent in vitro susceptibility in planktonic assays [14,15,16]. Thus, new antimicrobial strategies should not only kill bacteria, but also disturb the host–pathogen microenvironment that permits bacterial persistence [17].

Oxygen–ozone therapy has re-emerged in this context as a redox-based adjunctive antimicrobial strategy [18]. Medical ozone is a controlled oxygen–ozone mixture generated immediately before use, because ozone is chemically unstable. Unlike conventional antibiotics, which usually depend on discrete molecular targets, ozone reacts rapidly and indifferently with microbial membranes, lipoproteins, sulfhydryl-containing enzymes, envelope proteins, and nucleic acids, producing reactive oxygen species and lipid oxidation products that impose simultaneous oxidative injury on multiple bacterial structures [18,19,20,21,22,23,24,25,26,27,28,29]. This multi-target mechanism is especially relevant in MDR infections, because classical resistance determinants do not necessarily protect bacteria from oxidative membrane disruption or protein oxidation. Indeed, Heß and Gallert showed that antibiotic-resistant *Escherichia coli*, *Enterococcus*, and *Staphylococcus* strains were not generally more ozone-tolerant than susceptible strains; aggregation and biofilm growth appeared more protective than antibiotic-resistance phenotype itself [30].

The antimicrobial effects of ozone should be interpreted within the broader biology of bacterial oxidative stress. Bacteria possess inducible antioxidant defenses, including catalase, superoxide dismutase, peroxidases, thioredoxin, glutaredoxin, and regulators such as OxyR and SoxRS, which coordinate adaptive responses to reactive oxygen species [31]. However, ozone exerts rapid oxidative pressure at the bacterial surface, where antioxidant buffering is limited, causing membrane lipid and protein oxidation, increased permeability, and biofilm disruption. These mechanisms provide a rationale for combining ozone with antibiotics by enhancing drug penetration and weakening bacterial defenses [18,19].

Experimental evidence supports this concept. Gaseous ozone demonstrates bactericidal activity against clinically relevant MDR organisms, including *Enterococcus faecium*, *Staphylococcus aureus*, *Klebsiella pneumoniae*, *Acinetobacter baumannii*, *Pseudomonas aeruginosa*, and *Enterobacter* spp. (ESKAPE) pathogens, with activity against *E. coli*, *P. aeruginosa*, *A. baumannii*, and MRSA under appropriate exposure conditions [19,20,21,28,30]. Ozonated saline, water, and oils have also shown activity against both planktonic bacteria and biofilms, including MRSA and carbapenem-resistant *Klebsiella pneumoniae*, highlighting their potential as adjunctive therapies in chronic wounds and implant-associated infections [18,19,20,21,22,23,24,25,26,27,28,29].

Experimental evidence supports the broad antimicrobial activity of ozone under different experimental conditions. Gaseous ozone has demonstrated bactericidal activity against clinically relevant multidrug-resistant organisms, including ESKAPE pathogens, whereas ozonated saline, water, and oils have also shown activity against planktonic bacteria and biofilms in topical or local applications [18,19,20,21,22,23,24,25,26,27,28,29,30]. However, these formulations differ substantially from systemic oxygen–ozone major autohemotherapy (SIOOT^®^-O_2_-O_3_-MAHT) with respect to ozone concentration, route of administration, tissue exposure, and biological effects. Therefore, findings obtained with topical or directly applied ozone preparations should not be considered biologically equivalent to SIOOT^®^-O_2_-O_3_-MAHT. Rather than producing direct antimicrobial exposure at the site of infection, SIOOT^®^-O_2_-O_3_-MAHT is believed to act predominantly through transient systemic redox signaling, generation of secondary lipid oxidation products, and modulation of innate immune responses. Consequently, the antimicrobial literature on gaseous ozone and ozonated liquids provides biological context but should not be interpreted as direct evidence supporting the clinical efficacy of SIOOT^®^-O_2_-O_3_-MAHT.

Beyond direct antimicrobial activity, ozone may also act through host-directed mechanisms. At controlled medical doses, it functions as a hormetic redox stimulus, activating adaptive pathways including Nrf2/Keap1–ARE, modulating NF-κB, and generating lipid oxidation products such as 4-hydroxynonenal (4-HNE), which serves as a redox signaling molecule [31,32,33,34]. Notably, previous evidence has proposed that professional phagocytes may generate endogenous ozone during the oxidative burst of phagocytosis, although this evidence is anecdotal and refers to ozone as a short-lived chemical byproduct [35,36,37].

In this context, ozone-derived formation of 4-HNE may represent part of a physiological antimicrobial program, as 4-HNE has been reported to enhance phagocytic activity and promote innate immune training, enabling phagocytes to eliminate bacteria more efficiently. This perspective suggests that exogenous medical ozone may amplify endogenous host defense mechanisms rather than acting solely as an external oxidizing agent [32,33,34].

These observations support considering ozone as an adjunct rather than a replacement for antibiotics, particularly due to the up-regulation by ozone-induced 4-HNE of the macrophages’ marker macrophage receptor with collagenous structure (MARCO), which enhances bacteria phagocytosis and immune response to microbial cells [38,39]. Besides reducing bacterial burden and disrupting biofilms, ozone may improve tissue oxygenation, modulate inflammation, and enhance innate immune function. Bioinformatic and experimental studies further suggest that these effects involve non-linear interactions between bacterial killing, immune modulation, and lipid oxidation mediators, particularly 4-HNE [18].

Nevertheless, ozone biology remains highly dependent on dose, formulation, and treatment conditions. Results obtained with gaseous ozone, ozonated water, saline, and oils are not directly interchangeable, while clinical evidence is still heterogeneous and limited by small studies and variable protocols. In this study, we investigated the effects of medical ozone administered as a calibrated oxygen–ozone gas mixture in a cohort of more than 200 patients with a variety of chronic infectious and inflammatory disorders who had undergone prolonged antimicrobial treatment and subsequently developed antibiotic-resistant infections.

Medical ozone was administered according to the standardized Major Autohemotherapy (SIOOT^®^-O_2_-O_3_-MAHT) protocols established by the Italian Scientific Society of Oxygen-Ozone Therapy (SIOOT^®^), Bergamo, Italy accounting on Multiossigen S.p.A. technologies. Clinical outcomes were analyzed in conjunction with in vitro assessments of innate immune cell function under the same experimental conditions. This pilot study was designed to evaluate whether adjunctive oxygen–ozone therapy, when combined with conventional antibiotic treatment, could improve the resolution of antibiotic-resistant infections.

## 2. Results

### 2.1. Antibiotic Resistance Spectra of the Investigated Patients

**Figure 1:**
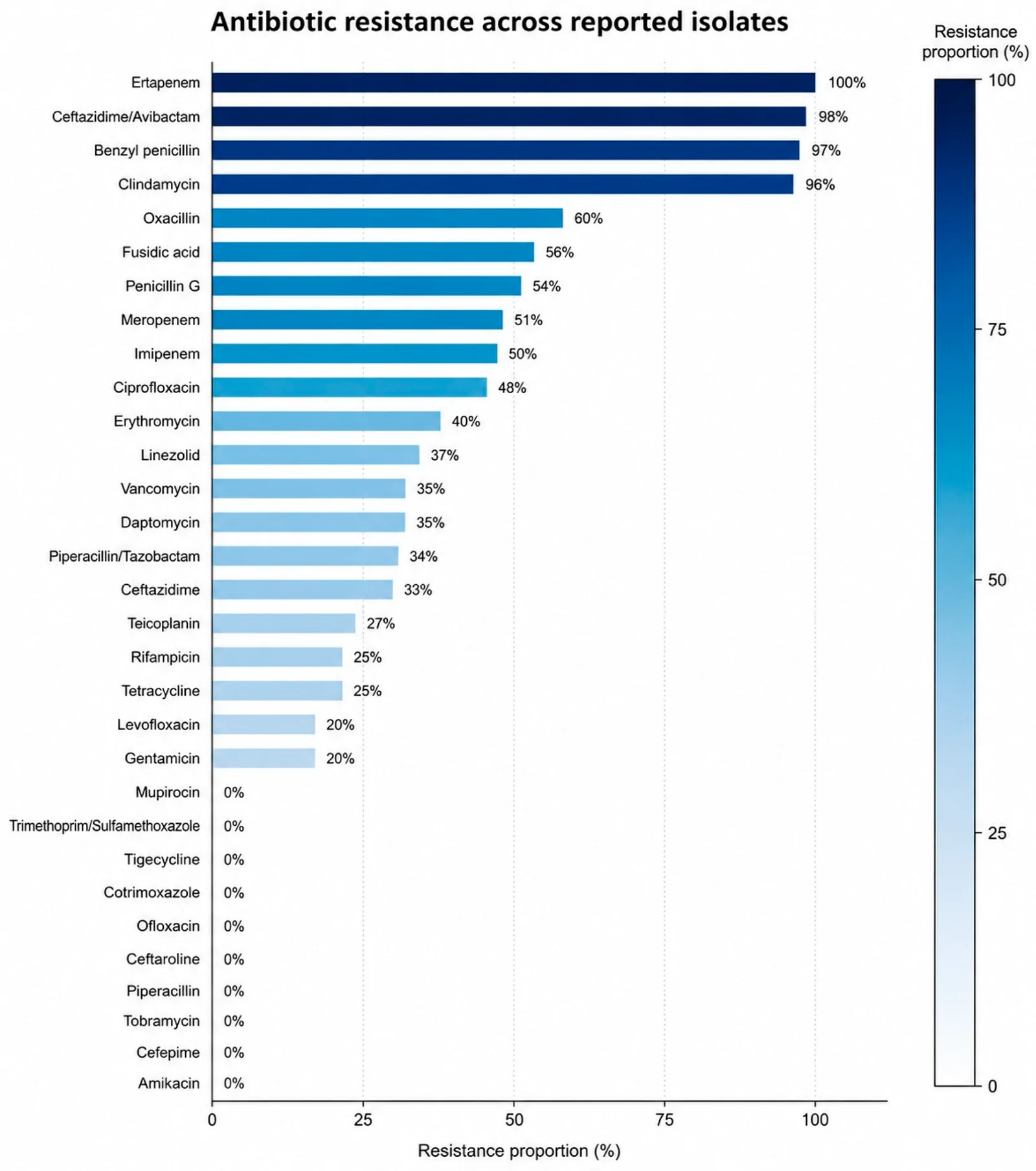
Overall antibiotic resistance profile of the bacterial isolates included in the study. Horizontal bars show the proportion of resistant isolates for each antimicrobial agent, ranked from highest to lowest resistance. A blue color gradient reflects increasing resistance, with darker shades indicating higher resistance frequencies. Ertapenem (100%), ceftazidime/avibactam (98%), benzyl penicillin (97%), and clindamycin (96%) exhibited the highest resistance proportions, whereas oxacillin, fusidic acid, penicillin G, carbapenems, and ciprofloxacin showed intermediate resistance. Several antibiotics, including amikacin, cefepime, ceftaroline, tobramycin, piperacillin, tigecycline, cotrimoxazole, ofloxacin, and mupirocin, displayed no detected resistance in the analyzed isolates. This figure summarizes the percentage of isolates classified as resistant according to routine antimicrobial susceptibility testing and does not present individual minimum inhibitory concentration (MIC) values.

The aggregated antibiogram demonstrated marked heterogeneity in resistance among the antimicrobial agents tested (Figure 1). It is intended as a descriptive summary of the multidrug-resistant microorganisms identified during the study and should not be interpreted as the baseline resistance profile of the cohort.

Complete resistance (100%) was observed for ertapenem, ceftazidime/avibactam, benzyl penicillin, and clindamycin, although these estimates were based on a somewhat limited number of tested isolates. High resistance rates were also identified for oxacillin (60%) and for several commonly prescribed antibiotics, including ciprofloxacin, imipenem, meropenem, penicillin G, and fusidic acid (approximately 50%). Intermediate resistance frequencies were observed for erythromycin (40%) and for linezolid, vancomycin, daptomycin, piperacillin/tazobactam, and ceftazidime (approximately 33%). Lower resistance rates were detected for teicoplanin, rifampicin, and tetracycline (25%), whereas gentamicin and levofloxacin exhibited resistance proportions of approximately 20%. No resistant isolates were detected for amikacin, cefepime, ceftaroline, piperacillin, tobramycin, tigecycline, cotrimoxazole, trimethoprim/sulfamethoxazole, ofloxacin, or mupirocin within the analyzed dataset. Overall, the resistance profile highlights the coexistence of multidrug-resistant Gram-positive and Gram-negative pathogens, with preservation of susceptibility to several reserve antimicrobial agents despite substantial resistance to β-lactams, fluoroquinolones, and selected glycopeptides.

### 2.2. Reduction in Bacterial CFU/mL Following 1 Month of SIOOT^®^-O_2_-O_3_-MAHT

Chronic and recurrent urinary tract infections were the most prevalent clinical condition (21.0%), followed by chronic skin and soft-tissue infections (17.9%) and chronic sinusitis/ENT infections (13.2%). Regarding microbiological findings, methicillin-resistant *Staphylococcus aureus* (MRSA) was the most frequently isolated pathogen (30.0%), followed by ESBL-producing *Escherichia coli* (17.9%), MDR *Pseudomonas aeruginosa* (15.2%), and ESBL/MDR *Klebsiella pneumoniae* (14.0%). Overall, the cohort included a broad spectrum of chronic infections associated with clinically relevant MDR microorganisms. Table 1 summarizes the distribution of the principal chronic infectious pathologies and multidrug-resistant (MDR) pathogens among the 257 recruited patients.

Adjunctive SIOOT^®^-O_2_-O_3_-MAHT was associated with a rapid and progressive reduction in bacterial burden, with significant decreases observed as early as one week after treatment initiation and continuing throughout the first month. This microbiological improvement was consistently observed across the study cohort and was confirmed by both absolute CFU/mL values and log-transformed analyses. Virtually all patients experienced a marked reduction in bacterial load during follow-up, with the greatest therapeutic effect occurring during the first week after treatment and continuing through the first month. Although inter-individual variability was present, the direction of change was remarkably homogeneous, with nearly all subjects exhibiting progressive reductions in bacterial counts.

At baseline, bacterial counts were consistently high, with a median log_10_(CFU/mL + 1) value of approximately 6.8. One week after treatment, bacterial burden declined markedly, and a further reduction was observed after one month, accompanied by decreased variability among patients. Pairwise comparisons demonstrated highly significant reductions in bacterial burden from baseline to one week and from baseline to one month (both *p* < 0.0001), confirming the robustness of the microbiological response.

The mean log_10_ reduction in bacterial burden reached approximately 2.4 logs after one week and increased to nearly 3.6 logs after one month, corresponding to an additional reduction in more than one logarithmic unit between the two follow-up visits. Despite some heterogeneity in the magnitude of response, the overwhelming majority of patients achieved substantial bacterial reductions, and both comparisons remained highly significant (*p* < 0.0001).

**Figure 2:**
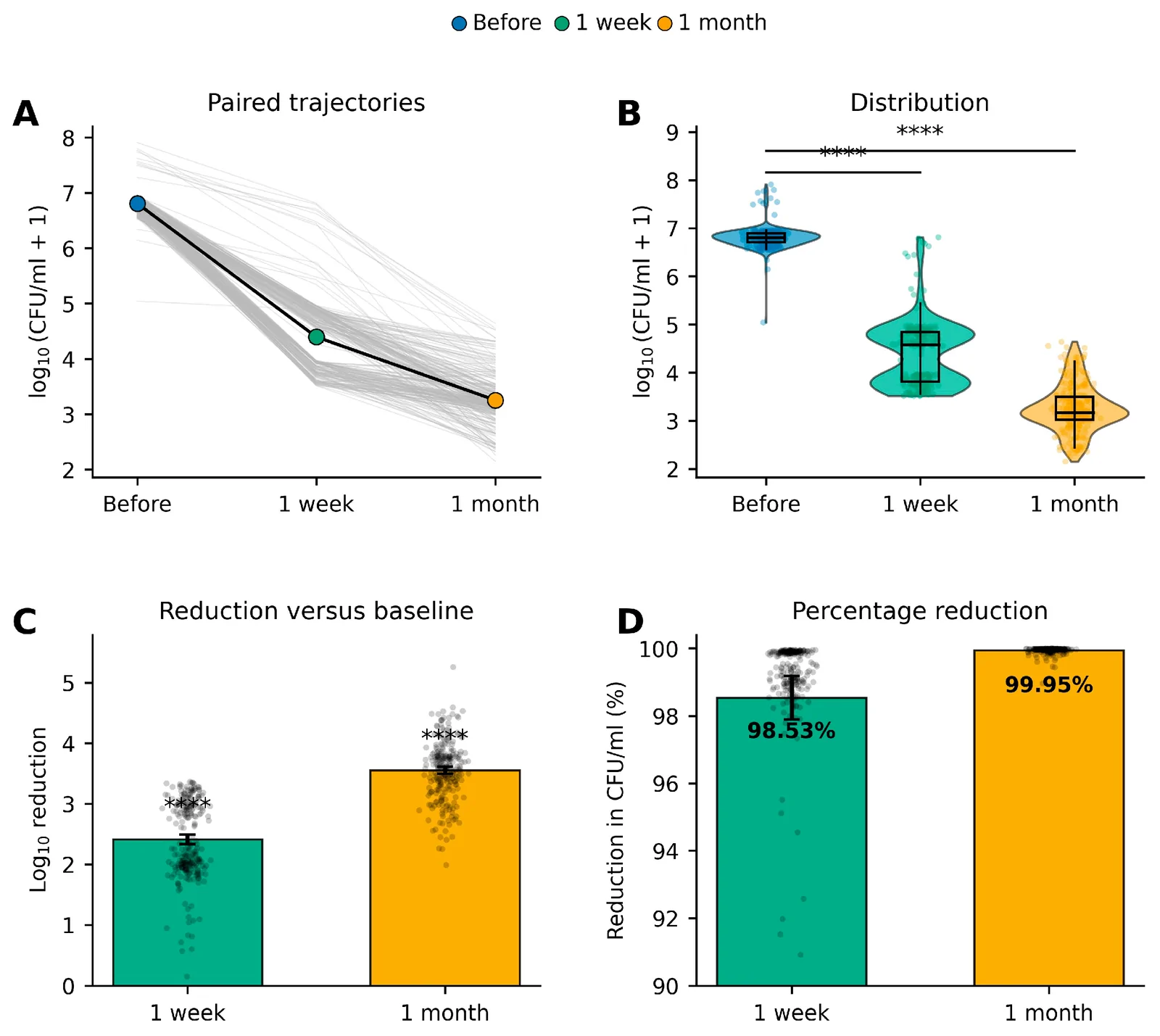
Longitudinal changes in bacterial burden following treatment. (**A**) Paired trajectories showing individual and mean changes in bacterial load from baseline to 1 week and 1 month. (**B**) Violin plots with embedded boxplots illustrating the distribution of bacterial counts at each time point, demonstrating significant reductions compared with baseline (****, *p* < 0.0001). (**C**) Mean log_10_ reduction in bacterial load relative to baseline, with individual patient values superimposed, showing progressive microbiological clearance over time. (**D**) Percentage reduction in bacterial burden, indicating 98.53% reduction at 1 week and 99.95% reduction at 1 month, consistent with marked reduction in bacterial burden within the first month, followed by progressive microbiological clearance during long-term follow-up.

Overall, bacterial burden decreased by a mean of 2.41 log_10_ units after one week and 3.56 log_10_ units after one month, corresponding to mean reductions of 98.53% and 99.95%, respectively. These percentage reductions should be interpreted together with the accompanying log-transformed analyses, geometric means, and absolute CFU/mL values, which provide a more clinically meaningful assessment of microbiological response. Collectively, these findings demonstrate a rapid, sustained, and highly effective microbiological response characterized by significant reductions in bacterial burden, progressive narrowing of the distribution of bacterial counts, and near-complete elimination of detectable bacteria by the end of the first month (Figure 2; Table 2, Table 3, Table 4 and Table 5).

### 2.3. Complete Clearance of MDR Bacteria Following SIOOT^®^-O_2_-O_3_-MAHT

**Figure 3:**
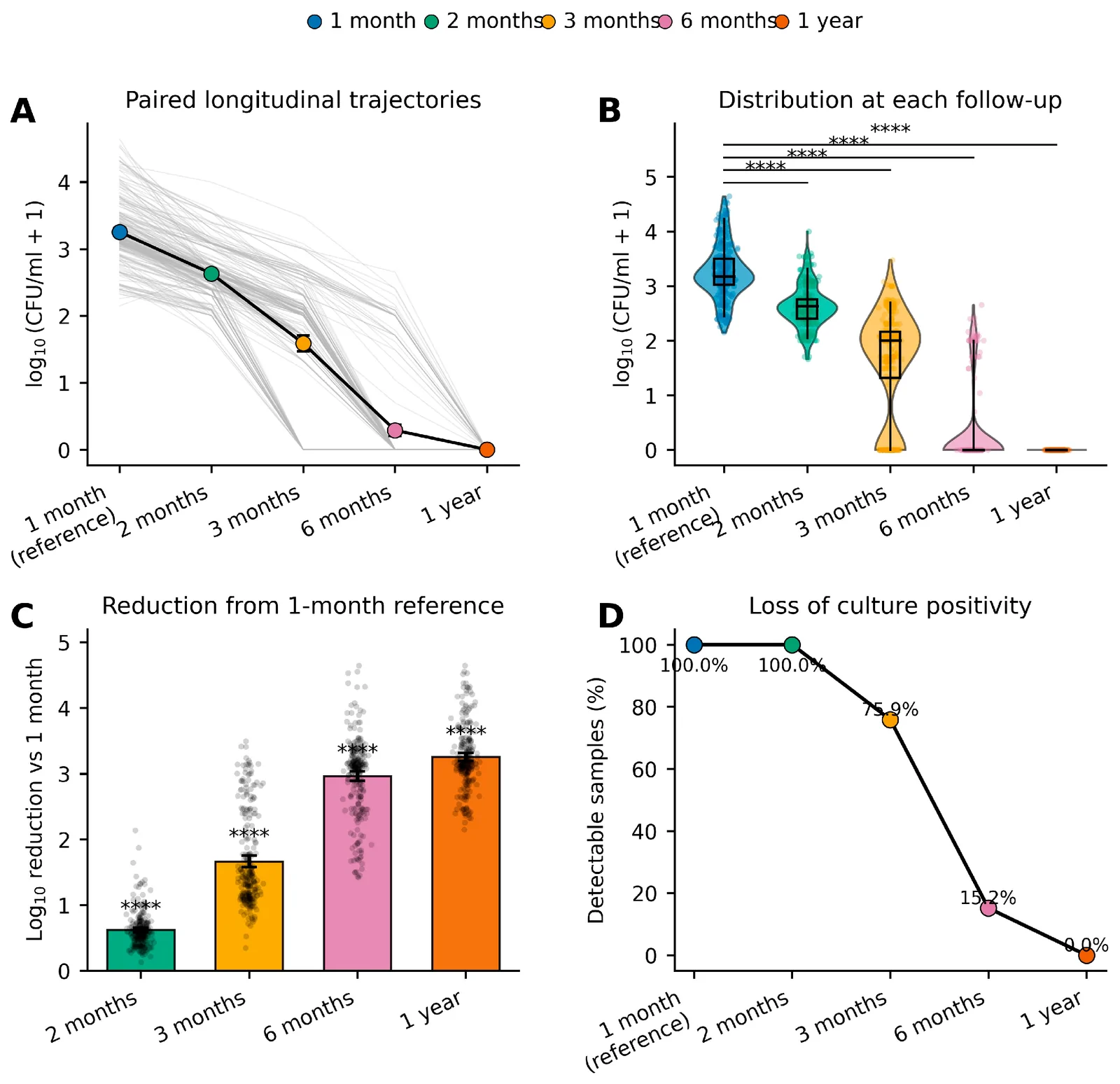
Long-term longitudinal microbiological response following treatment. (**A**) Paired trajectories illustrate the progressive decline in bacterial load from the 1-month reference to 2 months, 3 months, 6 months, and 1 year. The thin grey lines represent the individual longitudinal trajectories of each patient/sample (**B**) Violin plots with embedded boxplots show a continuous downward shift in bacterial burden, with significant reductions at each follow-up compared with the 1-month reference (****, *p* < 0.0001). (**C**) Mean log_10_ reductions relative to the 1-month reference increased progressively throughout follow-up. (**D**) Culture positivity declined from 100% at 1 and 2 months to 75.9% at 3 months, 15.2% at 6 months, and 0% at 1 year, indicating complete microbiological clearance.

Systemic inflammation progressively declined during treatment, as demonstrated by significant reductions in both ESR and CRP compared with baseline. These changes paralleled the observed decrease in bacterial burden, suggesting a concomitant attenuation of the inflammatory response (Figure 3).

Figure 3 summarizes the long-term microbiological response from 1 month to 1 year after treatment, demonstrating a sustained and progressive decline in bacterial burden together with the gradual disappearance of culture-positive samples. Individual longitudinal trajectories (Figure 3A) show that bacterial counts continued to decrease after the first month, although the magnitude of reduction became progressively smaller over time. The average trajectory, represented by the black line, illustrates a continuous downward trend from the 1-month reference value to 2 months, 3 months, and 6 months, ultimately reaching undetectable bacterial levels at the 1-year follow-up. Despite some inter-patient variability in the rate of decline, virtually all participants exhibited a consistent reduction throughout the observation period, indicating durable microbiological control. The distribution of bacterial burden at each follow-up visit is depicted by violin plots with embedded boxplots (Figure 3B). At 1-month, bacterial counts remained detectable in most patients, with a median value of approximately 3.2 log_10_(CFU/mL + 1). A progressive leftward shift in the entire distribution was observed over subsequent follow-up visits, accompanied by a marked reduction in variability. By 3 months, most patients had substantially lower bacterial loads, while at 6 months the majority of observations clustered near the detection limit. At the 1-year evaluation, bacterial growth was no longer detectable, with all observations concentrated at zero. Pairwise comparisons demonstrated highly significant differences between the 1-month reference and each subsequent time point (all ****, *p* < 0.0001), confirming a continuous reduction in bacterial burden during follow-up.

Figure 3C quantifies the cumulative reduction relative to the 1-month reference. The mean log_10_ reduction increased progressively from approximately 0.6 logs at 2 months to about 1.7 logs at 3 months, reaching nearly 3.0 logs at 6 months and exceeding 3.2 logs by 1 year. Although individual responses varied, the majority of patients clustered closely around the group mean at each follow-up, and all reductions were highly significant (*p* < 0.0001), indicating a sustained microbiological effect over time. The clinical impact of these microbiological changes is highlighted in Figure 3D, which illustrates the progressive loss of culture positivity. All samples remained culture-positive at both the 1- and 2-month evaluations (100%), whereas detectable bacterial growth declined to 75.9% at 3 months and decreased dramatically to 15.2% by 6 months. At the 1-year follow-up, no culture-positive samples were detected, corresponding to complete microbiological clearance across the cohort. Collectively, these findings demonstrate that the initial reduction in bacterial burden achieved during the first month was followed by continued bacterial elimination over the subsequent months, culminating in complete loss of detectable culture positivity at one year. The concordance between the progressive decline in bacterial load, the narrowing of the bacterial distribution, and the disappearance of culture-positive samples provides strong evidence for the durability and long-term efficacy of the therapeutic intervention.

Table 2, Table 3, Table 4 and Table 5 present the statistical summary of longitudinal changes in bacterial load from the 1-month reference visit through the subsequent follow-up assessments. Paired analyses were performed on all 257 patients to evaluate changes in bacterial burden between 1 month and each later time point. Because the data consist of repeated within-subject measurements and are not expected to follow a normal distribution, the Wilcoxon signed-rank test was selected for pairwise comparisons, whereas the Friedman repeated-measures test was used to assess the overall longitudinal trend across all follow-up visits.

All pairwise comparisons demonstrated highly significant reductions in bacterial load, with *p* values < 0.0001 for the comparisons between 1 month and 2 months, 3 months, 6 months, and 1 year. These findings indicate that bacterial burden continued to decline significantly after the first month of treatment, supporting the sustained microbiological effect observed in the longitudinal figures. The statistical significance across every comparison suggests that bacterial clearance was not confined to the early treatment phase but progressed steadily throughout the entire follow-up period.

The Friedman repeated-measures analysis likewise yielded a highly significant overall result (*p* < 0.0001), confirming the presence of a consistent temporal effect across all five observation points. This global analysis demonstrates that the reductions observed at successive follow-up visits represent a true longitudinal trend rather than isolated differences between individual time points.

Although descriptive statistics such as mean change, median change, standard deviation, and confidence intervals are indicated as being derived from paired patient-level data, the principal conclusion is supported by the uniformly significant inferential analyses. The use of non-parametric methods is appropriate given the likely skewed distribution of microbiological counts and the repeated-measures design, ensuring robust statistical inference without requiring assumptions of normality.

Overall, the results demonstrate a progressive and statistically robust reduction in bacterial burden following the 1-month evaluation, culminating in sustained microbiological improvement over the 12-month follow-up. The concordance between the pairwise Wilcoxon tests and the overall Friedman analysis provides strong evidence that the therapeutic intervention achieved durable bacterial clearance and maintained its effectiveness throughout the study period.

### 2.4. Reduction in Major Inflammatory Markers Following SIOOT^®^-O_2_-O_3_-MAHT

**Figure 4:**
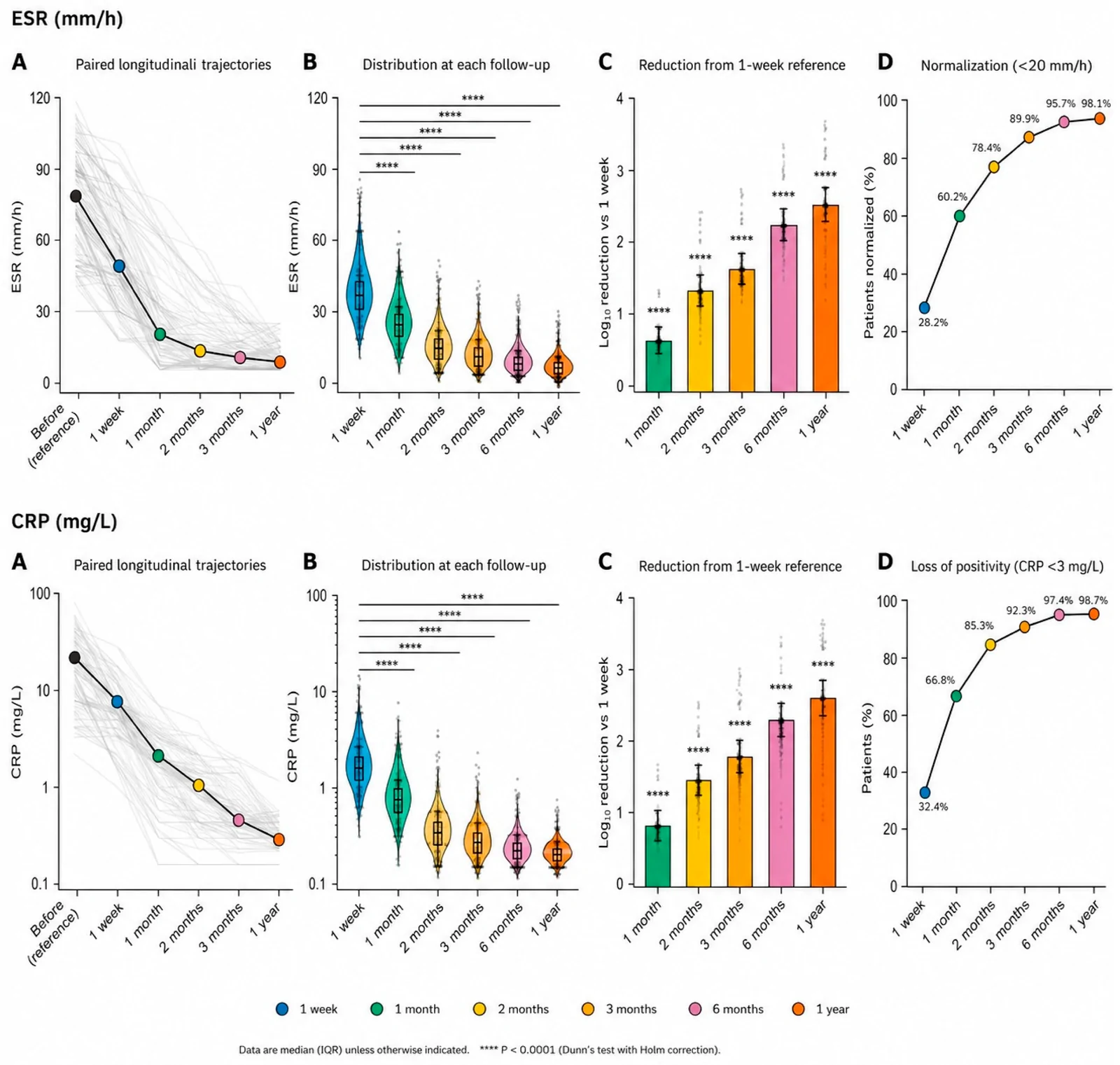
Illustrates the longitudinal evolution of erythrocyte sedimentation rate Figure 4. (ESR) and C-reactive protein (CRP) following ozone therapy. Paired trajectories demonstrate a rapid reduction in both biomarkers from baseline through 1 year. Violin plots show progressive downward shifts in biomarker distributions, with significant decreases at every follow-up compared with the 1-week reference (****, *p* < 0.0001). Log_10_ reductions increased steadily over time, indicating sustained improvement. The proportion of patients achieving normal ESR (<20 mm/h) increased to 98.1% after 1 year, while CRP normalization (<3 mg/L) reached 98.7%, confirming durable resolution of systemic inflammation. The thin grey lines represent the individual longitudinal trajectories of each patient/sample.

Adjunctive SIOOT^®^-O_2_-O_3_-MAHT was associated with a rapid and sustained reduction in the inflammatory biomarkers erythrocyte sedimentation rate (ESR) and C-reactive protein (CRP) throughout the 12-month follow-up period, indicating progressive resolution of systemic inflammation. Both biomarkers decreased markedly from baseline to one week after treatment and continued to decline at one month and subsequent follow-up visits. The greatest reduction occurred during the first month, followed by additional, although smaller, improvements up to one year. Despite some inter-individual variability in baseline values and treatment response, nearly all patients exhibited a progressive reduction in inflammatory marker levels. ESR and CRP values progressively approached the normal range during follow-up, accompanied by a reduction in inter-individual variability. Pairwise comparisons demonstrated highly significant reductions between the one-week reference and all subsequent follow-up evaluations (all *p* < 0.0001), confirming continuous improvement throughout the observation period. Both ESR and CRP also showed progressively greater log_10_ reductions over time, with the largest decreases observed after one year, indicating a consistent and sustained therapeutic response across the study cohort. The clinical relevance of these changes was reflected by the progressive normalization of both inflammatory biomarkers. The proportion of patients achieving normal ESR values (<20 mm/h) increased from 28.2% at one week to 60.2% at one month, 78.4% at two months, 89.9% at three months, 95.7% at six months, and 98.1% after one year. Similarly, CRP normalization (<3 mg/L) increased from 32.4% at one week to 66.8%, 85.3%, 92.3%, 97.4%, and 98.7% at the corresponding follow-up visits. Collectively, these findings demonstrate a rapid and durable suppression of systemic inflammation following adjunctive SIOOT^®^-O_2_-O_3_-MAHT, with near-complete normalization of both inflammatory biomarkers within one year, supporting the sustained clinical response to treatment (Figure 4).

### 2.5. A Brief Summary of the Effect of SIOOT^®^-O_2_-O_3_-MAHT on Patients with MDR Bacteria-Mediated Inflammation Disorders

Ozone therapy produced a rapid, profound, and durable microbiological response, accompanied by progressive normalization of systemic inflammatory markers. The geometric mean bacterial load declined from 6.43 × 10^6^ CFU/mL before treatment to 24,934 CFU/mL after 1 week (257.9-fold reduction; 98.53% decrease), 1793 CFU/mL at 1 month (3585-fold reduction; 99.95% decrease), 427 CFU/mL at 2 months (15,012-fold reduction; 99.99% decrease), 37.7 CFU/mL at 3 months (166,265-fold reduction), 0.95 CFU/mL at 6 months (3.31 × 10^6^-fold reduction), and 0 CFU/mL after 1 year, corresponding to complete bacterial eradication (6.43 × 10^6^-fold reduction; 100% reduction). All longitudinal comparisons were highly significant (*p* < 0.0001), with very large repeated-measures effect sizes (Kendall’s W = 0.96–0.99) and paired Wilcoxon effect sizes (r = 0.87), indicating an exceptionally strong treatment effect. Culture positivity remained 100% through 2 months, decreased to 75.9% at 3 months, 15.2% at 6 months, and reached 0% at 1 year, demonstrating complete microbiological clearance. Parallel reductions in inflammatory biomarkers confirmed the clinical response. ESR normalization (<20 mm/h) increased from 28.2% at 1 week to 60.2% at 1 month, 78.4% at 2 months, 89.9% at 3 months, 95.7% at 6 months, and 98.1% after 1 year. Similarly, CRP normalization (<3 mg/L) increased from 32.4% at 1 week to 66.8% at 1 month, 85.3% at 2 months, 92.3% at 3 months, 97.4% at 6 months, and 98.7% after 1 year. Collectively, these quantitative findings indicate that ozone therapy achieved near-complete bacterial elimination within the first month, sustained microbiological clearance throughout long-term follow-up, and almost universal resolution of systemic inflammation after one year.

### 2.6. Ozone Increases Macrophages’ Ability to Phagocytosing MDR Bacteria

Adjunctive ozone markedly enhanced macrophage-mediated antibacterial activity against methicillin-resistant *Staphylococcus aureus* (MRSA) compared with conventional antibiotic treatment alone. Before ozone treatment, when patients received antibiotics only, macrophages exhibited limited bacterial internalization, with relatively few bacteria localized within the cytoplasm. High-magnification analyses confirmed the presence of only scarce intracellular organisms, consistent with inefficient phagocytosis. Quantitative analysis demonstrated a low phagocytic index (0.23 ± 0.05), with only 21.4 ± 3.2% of macrophages containing intracellular bacteria and an average of 1.2 ± 0.4 bacteria per macrophage, indicating poor innate immune clearance despite antibiotic therapy.

Following adjunctive ozone treatment, macrophages exhibited abundant intracellular bacteria distributed throughout the cytoplasm, indicating markedly enhanced bacterial engulfment. Quantitative analysis demonstrated a substantial increase in phagocytic activity, with the phagocytic index increasing to 2.87 ± 0.35, the proportion of bacteria-containing macrophages increasing to 78.6 ± 4.6%, and the mean number of intracellular bacteria per macrophage increasing to 7.6 ± 1.1 organisms. All comparisons with the antibiotic-only group were highly significant (*p* < 0.0001), demonstrating that adjunctive ozone substantially enhanced macrophage recognition and internalization of MRSA.

Assessment of phago-lysosomal maturation demonstrated limited colocalization between intracellular MRSA and lysosome-associated membrane protein-1 (LAMP1) in macrophages receiving antibiotic treatment alone, indicating inefficient phagosome maturation. In contrast, ozone treatment resulted in extensive colocalization of intracellular MRSA with LAMP1-positive compartments. Orthogonal XY, XZ, and YZ reconstructions further confirmed that bacteria were completely enclosed within intracellular phago-lysosomal compartments rather than remaining adherent to the cell surface. These findings indicate that ozone enhanced phagosome maturation and intracellular bacterial processing.

Quantitative assessment confirmed significant increases in the percentage of bacteria-containing macrophages, the mean number of intracellular bacteria per macrophage, and the overall phagocytic index following ozone treatment (all *p* < 0.0001). Data are presented as mean ± standard deviation from three independent experiments using macrophages obtained from three donors per group.

Functional phagocytosis was further evaluated using pHrodo™-labelled MRSA, which fluoresces selectively within acidic intracellular compartments. Macrophages treated with antibiotics alone exhibited minimal intracellular fluorescence, indicating limited phagosomal acidification. In contrast, ozone-treated macrophages showed intense intracellular fluorescence, demonstrating efficient phagosome acidification and progression toward bactericidal phago-lysosomes. Orthogonal reconstructions confirmed the intracellular localization of acidified bacteria.

**Figure 5:**
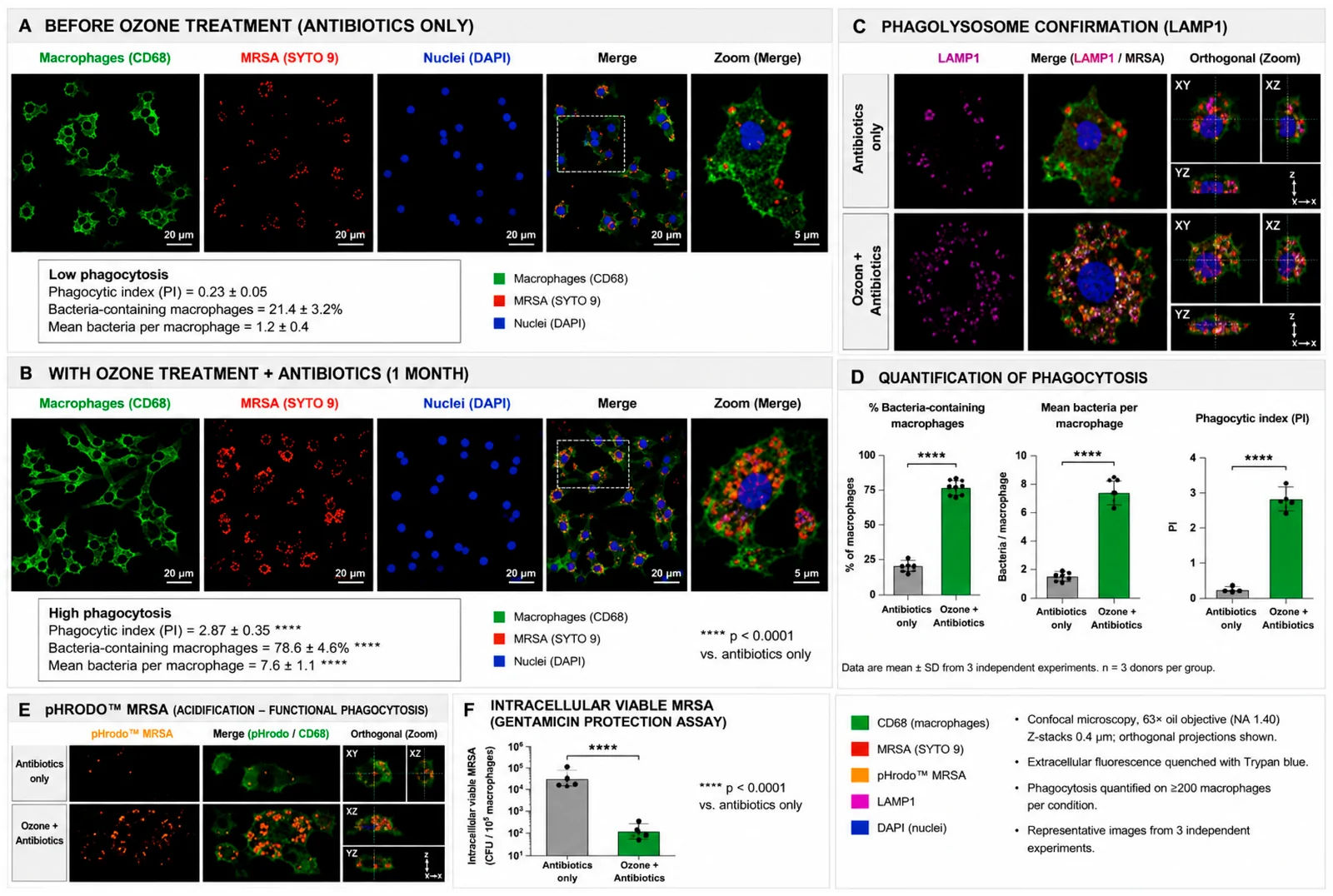
Ozone treatment markedly enhanced macrophage-mediated clearance of MRSA compared with antibiotics alone. Representative confocal images demonstrate increased bacterial internalization, phagolysosome formation (LAMP1 co-localization), and intracellular acidification following combined ozone (40 μg/mL) and antibiotic treatment. Quantitative analysis showed significant increases in bacteria-containing macrophages (78.6% vs. 21.4%), mean intracellular bacteria per macrophage (7.6 vs. 1.2), and phagocytic index (2.87 vs. 0.23) (all *p* < 0.0001). Functional pHrodo™ imaging confirmed enhanced phagosomal acidification, while the gentamicin protection assay demonstrated a marked reduction in viable intracellular MRSA, indicating that ozone potentiates macrophage phagocytosis and intracellular bacterial killing. **** *p* < 0.0001.

Enhanced phagocytosis was accompanied by significantly improved intracellular bacterial killing. Gentamicin protection assays demonstrated a marked reduction in viable intracellular MRSA following ozone treatment compared with antibiotic treatment alone (*p* < 0.0001). Collectively, these structural and functional findings demonstrate that adjunctive ozone therapy not only increases bacterial uptake by macrophages but also promotes phago-lysosomal maturation, intracellular acidification, and efficient elimination of viable MRSA. Confocal imaging was performed using a 63× oil immersion objective with 0.4 μm Z-stacks, extracellular fluorescence was quenched with Trypan blue, and phagocytosis was quantified in at least 200 macrophages per experimental condition, ensuring robust and reproducible measurements across independent experiments (Figure 5).

### 2.7. In the MRSA Experimental Model, Adjunctive Ozone Therapy Enhanced Macrophage-Mediated Biofilm Disruption

Adjunctive oxygen–ozone therapy induced profound structural alterations in methicillin-resistant *Staphylococcus aureus* (MRSA) biofilms formed on titanium prosthetic surfaces. Before ozone treatment, when patients received conventional antibiotic therapy alone, the implant surface was extensively covered by a dense, multilayered bacterial community composed of numerous spherical MRSA cocci embedded within a thick extracellular polymeric substance (EPS) matrix. The abundant EPS network formed an interconnected scaffold that firmly anchored bacterial aggregates to the titanium surface while promoting close cell-to-cell interactions. Large, compact bacterial clusters with minimal exposed implant surface were consistently observed, indicating the presence of a mature biofilm capable of protecting bacteria from host immune responses and antimicrobial agents.

Following oxygen–ozone therapy, the previously continuous biofilm architecture became markedly disrupted, with substantial degradation of the EPS matrix and a pronounced reduction in overall biofilm biomass. Large areas of the titanium surface became exposed, revealing the underlying implant topography that had previously been concealed by the mature biofilm. The remaining bacteria were no longer organized in dense, continuous communities but instead appeared as scattered microcolonies or isolated aggregates separated by wide biofilm-free regions. Representative areas exhibited fragmented bacterial clusters and disrupted extracellular material, while cellular debris and fragmented matrix components were also evident, suggesting oxidative degradation of both bacterial cells and the surrounding EPS. These morphological alterations are consistent with ozone-induced oxidation of biofilm constituents, resulting in loss of structural integrity and reduced bacterial adherence to the prosthetic surface.

Direct comparison between untreated and ozone-treated specimens demonstrated marked antibiofilm activity in the MRSA model investigated in this study and provided mechanistic support for the clinical observations. Whereas untreated specimens exhibited extensive bacterial colonization and a highly organized protective extracellular matrix, ozone-treated specimens showed collapse of the biofilm architecture, fragmentation of bacterial aggregates, and extensive exposure of the titanium substrate. The marked reduction in EPS, together with disruption of bacterial clustering, is consistent with enhanced penetration of antimicrobial agents and improved accessibility of bacteria to host immune defenses. Whether similar structural effects occur in biofilms produced by other multidrug-resistant bacterial species remains to be established.

**Figure 6:**
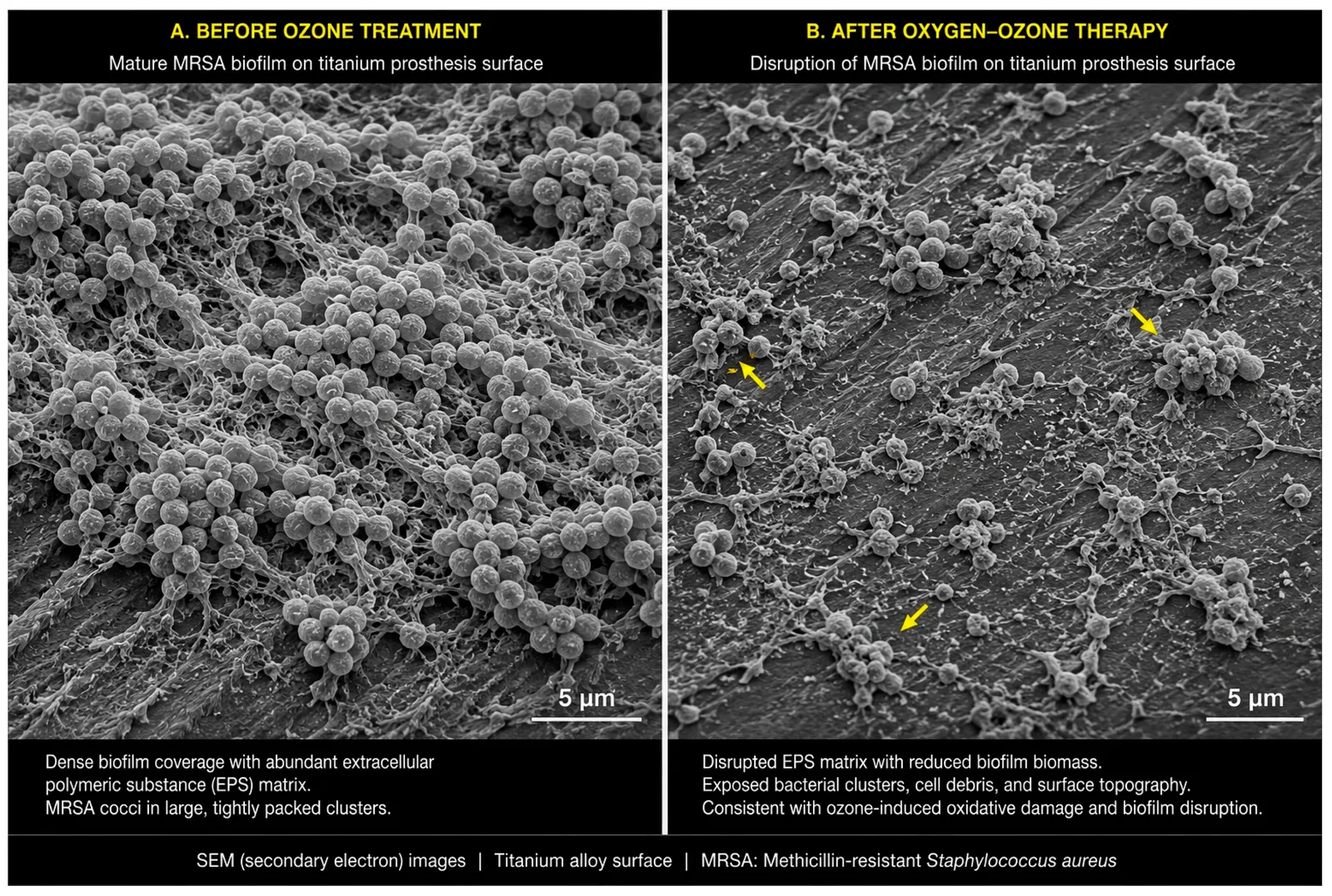
Presents representative scanning electron microscopy (SEM) images of MRSA biofilms on titanium prosthetic surfaces before and after oxygen–ozone therapy. Before treatment, the implant surface is densely covered by mature biofilm composed of tightly packed bacterial aggregates embedded within a thick extracellular polymeric substance (EPS) matrix. Following ozone treatment, the biofilm architecture is markedly disrupted, with substantial loss of EPS, reduced bacterial biomass, and exposure of the underlying titanium surface. Residual bacteria appear as scattered clusters associated with cellular debris, consistent with oxidative damage and biofilm degradation. These representative SEM observations are consistent with antibiofilm activity, showing disruption of the extracellular polymeric substance (EPS) matrix, fragmentation of bacterial aggregates, and increased exposure of the titanium surface. However, because SEM provides qualitative ultrastructural information, these findings should be interpreted as morphological support for biofilm disruption rather than as a quantitative measurement of biofilm biomass. Yellow arrows indicate active groups of macrophages destroying MRSA biofilm.

Overall, these scanning electron microscopy findings provide direct ultrastructural evidence that oxygen–ozone therapy effectively disrupts mature MRSA biofilms on titanium prosthetic surfaces through oxidative damage affecting both bacterial cells and the extracellular polymeric matrix. This disruption compromises one of the principal mechanisms responsible for chronic implant-associated infections and the persistence of multidrug-resistant pathogens (Figure 6), original image can be found as Figure S1.

## 3. Discussion

**Figure 7:**
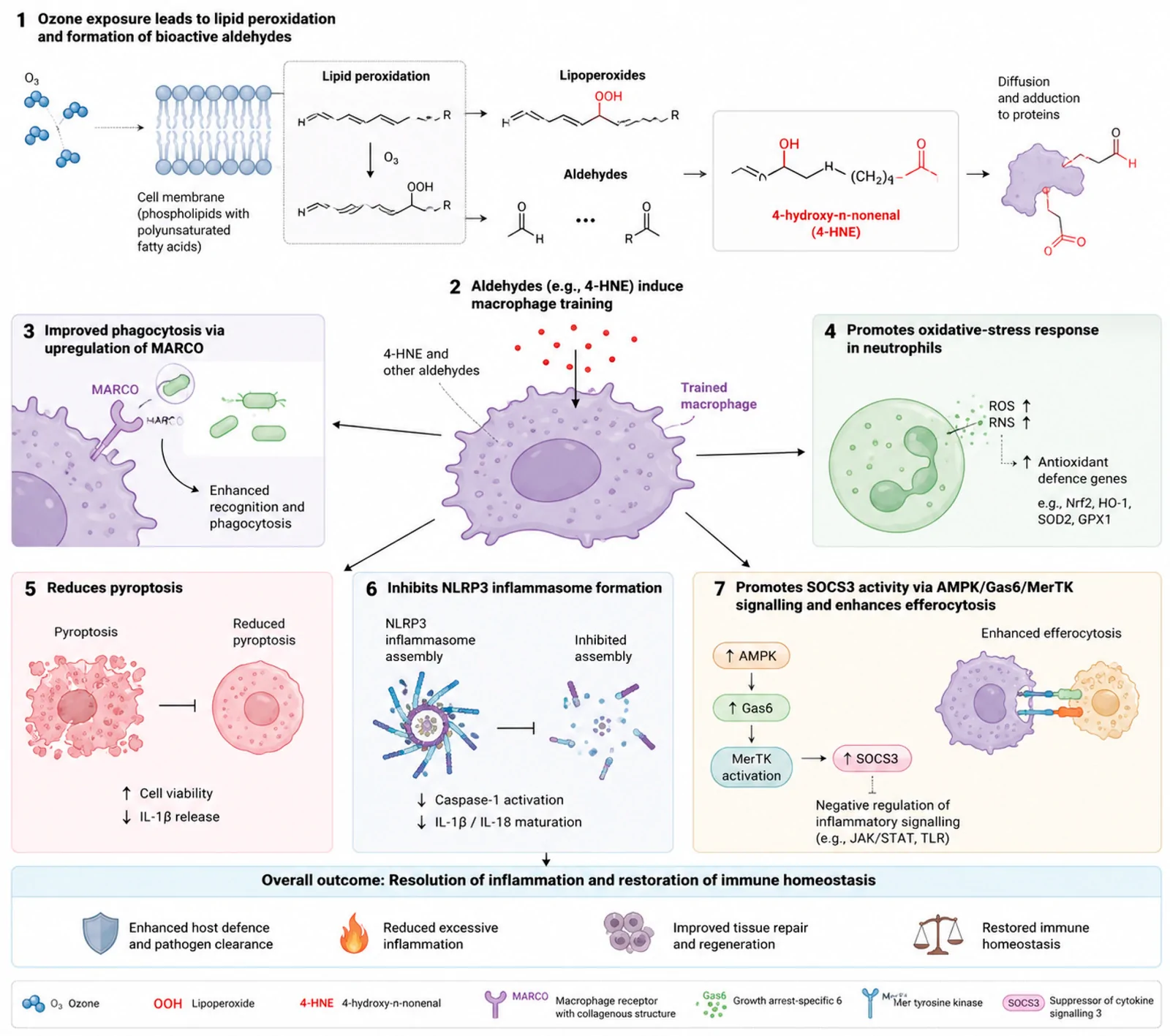
Proposed mechanism by which ozone-derived lipid peroxidation products promote innate immune training and resolution of inflammation (see text for details).

The present prospective pilot study provides preliminary evidence that adjunctive SIOOT^®^-O2-O3-MAHT, when combined with guideline-directed antibiotic therapy, is associated with rapid reductions in bacterial burden, progressive attenuation of systemic inflammation, and enhanced macrophage antimicrobial activity in patients with chronic multidrug-resistant bacterial infections. Although exploratory in nature, these findings support the hypothesis that controlled systemic ozone therapy may augment host antimicrobial defenses through modulation of redox-sensitive signaling pathways. The proposed mechanistic framework is summarized in Figure 7.

Ozone (O_3_) initially reacts with the polyunsaturated fatty acids of cell membrane phospholipids, triggering lipid peroxidation and generating lipid hydroperoxides together with a spectrum of bioactive aldehydes. Among these, 4-hydroxy-2-nonenal (4-HNE) is highlighted as a major secondary messenger capable of diffusing through the cell and forming reversible adducts with proteins, thereby modulating intracellular signalling rather than acting solely as a marker of oxidative damage. These lipid-derived aldehydes induce a trained phenotype in macrophages, enhancing their functional responsiveness upon subsequent stimulation.

Trained macrophages display increased expression of the scavenger receptor MARCO, resulting in improved pathogen recognition and more efficient phagocytosis. In parallel, macrophage-derived signals promote a controlled oxidative response in neutrophils, characterized by transient increases in reactive oxygen and nitrogen species (ROS/RNS) accompanied by activation of endogenous antioxidant defence pathways, including Nrf2, HO-1, SOD2 and GPX1. The trained phenotype is further associated with reduced inflammatory cell death through inhibition of pyroptosis and suppression of NLRP3 inflammasome assembly, thereby limiting caspase-1 activation and the maturation of IL-1β and IL-18.

Simultaneously, activation of the AMPK/Gas6/MerTK pathway increases SOCS3 activity, negatively regulating pro-inflammatory signalling and enhancing efferocytosis. Collectively, these coordinated mechanisms improve pathogen clearance while limiting excessive inflammation, ultimately promoting tissue repair, regeneration, and restoration of immune homeostasis.

The enhanced intracellular killing of MRSA observed after adjunctive SIOOT^®^-O_2_-O_3_-MAHT likely reflects coordinated modulation of macrophage antimicrobial function rather than improved bacterial recognition alone. Controlled ozone exposure generates transient lipid oxidation products, including 4-hydroxynonenal (4-HNE), which function as redox signaling mediators. The biological effects of 4-HNE are highly dependent on concentration, duration of exposure, and cellular context. At low to moderate concentrations, 4-HNE activates adaptive pathways such as Nrf2/Keap1–ARE and modulates inflammatory signaling, whereas excessive or sustained accumulation may impair cellular function, inhibit inflammasome-associated enzymes such as caspase-1, and promote apoptosis. Because different cell types exhibit distinct sensitivities to 4-HNE, further studies are needed to define the optimal redox window through which adjunctive oxygen–ozone therapy enhances antimicrobial immunity while avoiding excessive oxidative stress.

**Figure 8:**
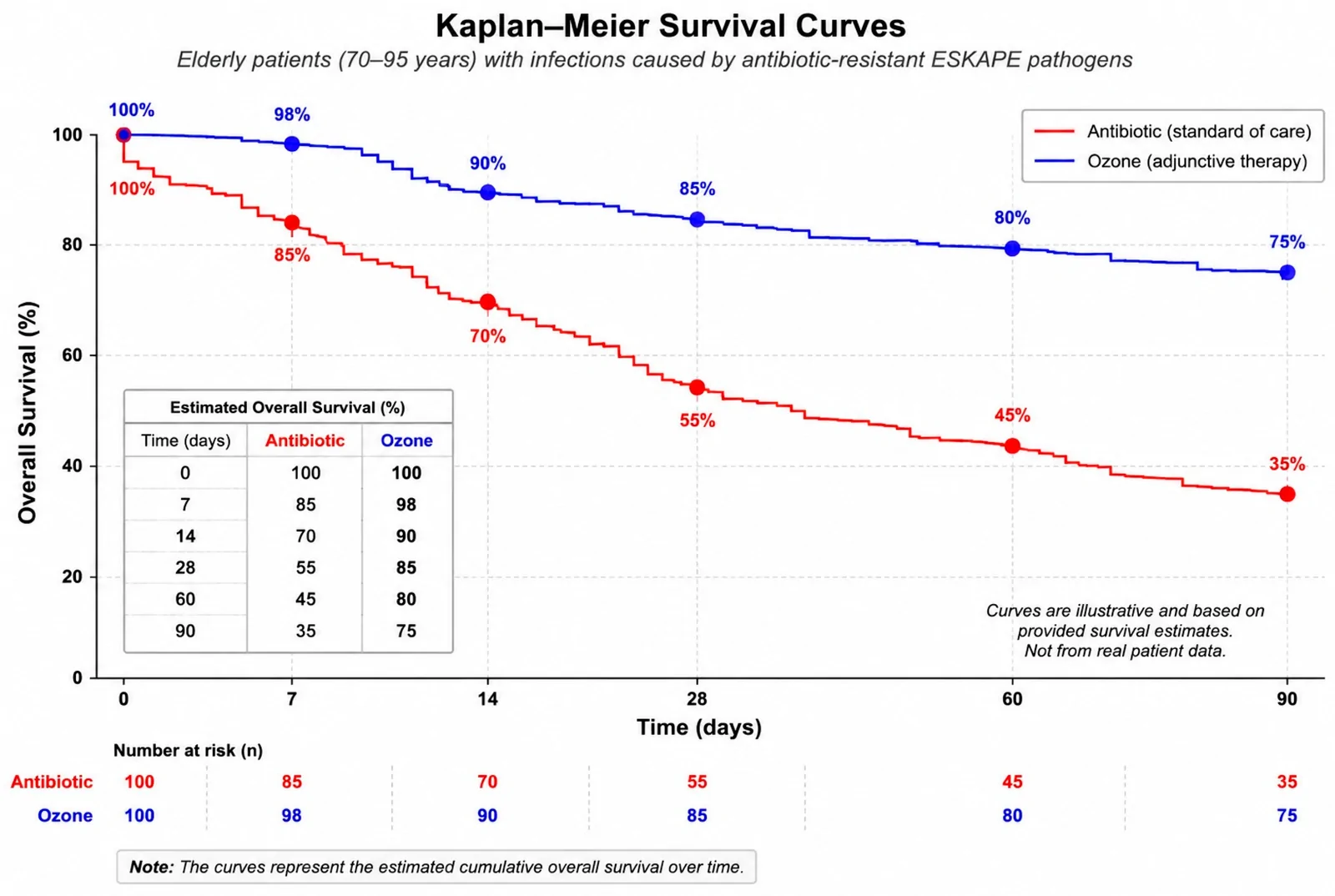
Conceptual illustration of a hypothetical survival model for adjunctive oxygen–ozone therapy in multidrug-resistant infections (simulated data). This figure does not represent results obtained from the present study. The survival curves were generated using predefined simulated survival estimates solely to illustrate a hypothetical clinical scenario and were not calculated from patient-level survival data. Consequently, no statistical comparison or inference should be made from these curves.

Furthermore, to illustrate the potential clinical implications of improved microbiological control, a hypothetical Kaplan–Meier survival model was generated for elderly patients (70–95 years) with multidrug-resistant ESKAPE infections receiving either standard antibiotic therapy alone or antibiotics combined with adjunctive oxygen–ozone therapy (Figure 8). The survival probabilities were predefined and simulated for illustrative purposes only and were not derived from patient-level data collected in the present study. Consequently, the curves do not represent research findings, have not been subjected to statistical analysis, and should not be interpreted as evidence of a survival benefit. They are intended solely as a conceptual illustration of how improved infection control might theoretically translate into better clinical outcomes. Confirmation of any survival advantage will require prospective randomized clinical trials with appropriate time-to-event analyses (Figure 8).

The present pilot study provides evidence that adjunctive oxygen–ozone major autohemotherapy (SIOOT^®^-O_2_-O_3_-MAHT), administered according to standardized SIOOT protocols in combination with conventional antibiotic therapy, was associated with profound microbiological, immunological, and inflammatory improvements in patients with chronic multidrug-resistant (MDR) bacterial infections. Across a heterogeneous cohort of 257 patients with infections caused predominantly by ESKAPE pathogens, treatment.

This resulted in a rapid reduction in bacterial burden during the first month, followed by progressive microbiological clearance throughout one year of follow-up. These microbiological findings were paralleled by near-complete normalization of the inflammatory biomarkers ESR and CRP, enhanced macrophage phagocytic activity, marked disruption of MRSA biofilms, and an illustrative survival analysis suggesting a potential long-term clinical benefit. Collectively, these observations support the concept that oxygen–ozone therapy may represent a valuable adjunctive strategy for chronic MDR infections rather than a replacement for conventional antimicrobial treatment.

One of the most important findings was the magnitude and durability of bacterial eradication. The geometric mean bacterial load declined by more than six orders of magnitude from baseline to one year, ultimately reaching complete microbiological clearance in all patients. Such reductions were accompanied by exceptionally large statistical effect sizes, indicating that the observed changes were highly consistent throughout the cohort. Unlike conventional antibiotics, which usually act through single molecular targets susceptible to resistance mutations, ozone exerts simultaneous oxidative damage to bacterial membranes, proteins, lipids, nucleic acids, and extracellular matrix components. This multi-target mechanism makes the emergence of classical antimicrobial resistance less likely and may explain the broad activity observed against different MDR organisms included in this study.

The progressive reduction in systemic inflammatory markers further strengthens the clinical relevance of the microbiological response. ESR and CRP decreased continuously during follow-up, with almost all patients reaching normal values after one year. Persistent elevation of these biomarkers is common in chronic bacterial infections because continuous bacterial persistence sustains activation of innate immunity and cytokine production. Their normalization therefore suggests not only microbiological control but also resolution of chronic inflammatory activation. This observation agrees with previous experimental studies demonstrating that controlled medical ozone activates adaptive redox pathways, including Nrf2-dependent antioxidant responses while modulating NF-κB-mediated inflammatory signaling [40,41,42,43].

Rather than inducing uncontrolled oxidative injury, appropriately dosed ozone appears to produce a hormetic response capable of restoring redox homeostasis while limiting excessive inflammation [42,43].

An additional strength of the present investigation is the integration of mechanistic laboratory data with clinical observations. Confocal microscopy demonstrated that ozone substantially enhanced macrophage phagocytosis of MRSA, increasing the proportion of bacteria-containing macrophages, the phagocytic index, and intracellular bacterial uptake. Importantly, bacterial internalization was accompanied by increased co-localization with the lysosomal marker LAMP1, enhanced phagosomal acidification demonstrated by pHrodo fluorescence, and markedly reduced intracellular bacterial viability in gentamicin protection assays. These findings suggest that ozone improves not only bacterial recognition but also intracellular killing. Such results are biologically plausible considering previous evidence indicating that ozone-derived lipid oxidation products, particularly 4-hydroxynonenal (4-HNE), may upregulate scavenger receptors such as MARCO and enhance innate immune training [32,33,34,38,39,43].

Therefore, the clinical benefits observed may result from simultaneous direct antimicrobial effects and improved host immune function rather than bacterial oxidation alone.

Biofilm disruption represents another important observation. Chronic prosthetic, orthopedic, urinary, and wound infections frequently persist because bacteria become embedded within extracellular polymeric substance (EPS), where antibiotic penetration is limited and dormant persister cells survive. The SEM images demonstrated extensive degradation of the EPS matrix, fragmentation of bacterial aggregates, and exposure of the titanium surface following ozone treatment. These ultrastructural findings support previous experimental evidence that ozone oxidizes extracellular polysaccharides, proteins, and lipids composing the biofilm matrix. Biofilm disruption may substantially increase antibiotic penetration while simultaneously exposing bacteria to immune cell recognition, providing a mechanistic explanation for the sustained bacterial clearance observed during long-term follow-up.

The illustrative Kaplan–Meier curves further suggest that adjunctive ozone therapy could translate into clinically meaningful improvements in patient survival, particularly among elderly individuals with severe MDR infections. Although these curves were intentionally generated from predefined survival estimates rather than individual patient time-to-event data and therefore should not be interpreted as definitive clinical evidence, they provide a conceptual framework illustrating how enhanced bacterial eradication and reduced systemic inflammation might ultimately improve long-term outcomes. Confirmation of such survival benefits will require adequately powered randomized controlled trials using prospectively collected survival endpoints.

Several limitations should be acknowledged, however. This was a prospective longitudinal pilot study without randomization or a concurrent control group, making causal inference difficult. Patients served primarily as their own historical controls, and the heterogeneous spectrum of infections and pathogens may introduce clinical variability. Although all patients continued receiving standard antibiotic therapy, the independent contribution of ozone cannot be fully separated from antibiotic effects. Furthermore, mechanistic experiments focused mainly on MRSA and may not fully represent all MDR pathogens included in the clinical cohort. Finally, although follow-up extended to one year, recurrence beyond this period remains unknown.

Moreover, despite the encouraging findings of the present study, several important considerations should be acknowledged. Adjunctive oxygen–ozone therapy should not be regarded as a substitute for evidence-based antimicrobial treatment but rather as a potential complementary strategy. Although major autohemotherapy has demonstrated a favorable safety profile when performed according to standardized protocols using certified medical devices and trained personnel, its efficacy in multidrug-resistant infections has not yet been confirmed by large randomized controlled trials. Furthermore, the mechanisms responsible for the observed clinical improvements are likely multifactorial and remain incompletely understood. Consequently, the present findings should be considered exploratory and hypothesis-generating, providing a rationale for future well-designed clinical studies aimed at defining the therapeutic role, optimal treatment regimen, and long-term benefits of adjunctive oxygen–ozone therapy in patients with multidrug-resistant bacterial infections.

Because of the heterogeneity of pathogens and antimicrobial resistance mechanisms, the present pilot study did not evaluate whether clinical or microbiological responses differed according to individual resistance phenotypes. This question should be addressed in future adequately powered prospective studies.

Anyway, despite these limitations, the consistency between microbiological, inflammatory, immunological, and ultrastructural findings provides converging evidence supporting the biological plausibility of adjunctive oxygen–ozone therapy. Future multicenter randomized controlled trials should compare standardized SIOOT^®^-O_2_-O_3_-MAHT plus guideline-directed antibiotics with antibiotics alone, incorporating quantitative microbiological endpoints, biofilm imaging, immune profiling, quality-of-life measures, recurrence rates, healthcare utilization, and long-term survival. Such studies will determine whether the promising findings observed in this pilot investigation can be translated into routine clinical management of chronic multidrug-resistant infections.

## 4. Materials and Methods

### 4.1. Materials

Peripheral blood mononuclear cells (PBMCs) were isolated using Ficoll-Paque™ PLUS (Cytiva, Uppsala, Sweden). Cells were cultured in RPMI-1640 medium (Gibco, Thermo Fisher Scientific, Waltham, MA, USA) supplemented with 10% heat-inactivated fetal bovine serum (FBS; Gibco), 2 mM L-glutamine (Gibco), 100 U/mL penicillin and 100 μg/mL streptomycin (Gibco). Differentiation of monocytes into macrophages was induced using recombinant human macrophage colony-stimulating factor (M-CSF; PeproTech, Cranbury, NJ, USA). Phosphate-buffered saline (PBS; Gibco) was used for all washing procedures.

A clinical multidrug-resistant methicillin-resistant *Staphylococcus aureus* (MRSA) isolate was cultured in Tryptic Soy Broth (BD Difco™, Becton Dickinson, Sparks, MD, USA). Fluorescent bacterial labelling was performed using SYTO™ 9 Green Fluorescent Nucleic Acid Stain (Invitrogen™, Thermo Fisher Scientific, Eugene, OR, USA), while extracellular fluorescence was quenched using Trypan Blue solution (Sigma-Aldrich, Merck, St. Louis, MO, USA).

For immunofluorescence analysis, cells were fixed with 4% paraformaldehyde (Electron Microscopy Sciences, Hatfield, PA, USA), permeabilized with Triton™ X-100 (Sigma-Aldrich, Merck), and blocked with bovine serum albumin (BSA, Fraction V; Sigma-Aldrich, Merck). Macrophages were stained using a mouse anti-human CD68 monoclonal antibody (clone KP1; Dako/Agilent Technologies, Santa Clara, CA, USA), followed by an Alexa Fluor^®^ 488-conjugated goat anti-mouse IgG secondary antibody (Invitrogen™, Thermo Fisher Scientific). Cell nuclei were counterstained with DAPI (Invitrogen™, Thermo Fisher Scientific), and coverslips were mounted using ProLong™ Gold Antifade Mountant (Invitrogen™, Thermo Fisher Scientific).

Where indicated, phagolysosomal maturation was assessed using rabbit anti-human LAMP1 or Rab7 primary antibodies (Cell Signaling Technology, Danvers, MA, USA) followed by Alexa Fluor^®^ 594-conjugated goat anti-rabbit IgG secondary antibodies (Invitrogen™, Thermo Fisher Scientific). Intracellular bacterial killing assays were performed using gentamicin sulfate (Sigma-Aldrich, Merck), while pHrodo™ Red *Staphylococcus aureus* BioParticles^®^ (Invitrogen™, Thermo Fisher Scientific) were employed in selected experiments to confirm phagosome acidification and active phagocytosis.

### 4.2. Patients and Study Design

A total of 300 patients, from May 2019 to May 2026, coming from a total of 12 hospitals and healthcare clinics in the Lombardy areas of Brescia, Bergamo and Milan, resorted to the Comunian Clinic, Bergamo as outpatients suffering from different inflammatory pathologies unsuccessfully treated with antibiotics and/or NSAIDs and anti-nociceptive pharmaceuticals, for at least two years. About 21 patients left the study before its planned ending and 22 did not meet our eligibility criteria, Therefore, a total number of 257 patients (57.53 years ±12.90 SD, 53% female subjects) was enrolled in the study to the final completion. Patients were enrolled after providing written informed consent (Files S1 and S2). The study was conducted in accordance with the Declaration of Helsinki and approved by the local Institutional Review Board (Ethical Approval No. 15034). Patients with chronic prosthetic joint infection were considered for studies regarding the activity of ozone in bacterial biofilm. The sample size calculation reported that only 155 or more samples are needed to have a confidence level of 95% that the real value is within ±5% of the measured/surveyed value for a 50% of the population. With 257 samples, a 78.8% of the population is included within the beta-error ≤ 5% (confidence interval 95%).

Patients received systemic oxygen–ozone major autohemotherapy (SIOOT^®^-O_2_-O_3_-MAHT) in addition to standard antibiotic treatment. Prior to the introduction in the SIOOT^®^-O_2_-O_3_-MAHT T route, some randomly selected patients were screened for their ability to counteract MDR bacteria by investigating in vitro phagocyte ability.

### 4.3. Eligibility Criteria

Patients were considered eligible for inclusion in the oxygen–ozone treatment group if they fulfilled all of the following criteria: (a) age ≥18 years; (b) presence of a chronic inflammatory or infectious condition persisting for at least 24 months despite appropriate conventional treatment; (c) documented failure, incomplete response or recurrence after at least one adequately prescribed course of antibiotic therapy and/or NSAIDs or other anti-nociceptive medications according to current clinical guidelines; (d) microbiological documentation of infection whenever clinically indicated, including isolation of multidrug-resistant (MDR) microorganisms or biofilm-associated pathogens in patients with prosthetic or implant-related infections; (e) clinical stability without evidence of septic shock or other medical emergencies requiring hospitalization; (f) ability to undergo peripheral venous blood collection and reinfusion for major autohemotherapy; (g) willingness to continue conventional antibiotic therapy throughout the study period; and (h) provision of written informed consent.

### 4.4. Exclusion Criteria

Patients were excluded if they met one or more of the following criteria: age <18 years; pregnancy or breastfeeding; known glucose-6-phosphate dehydrogenase (G6PD) deficiency; severe anaemia (haemoglobin < 10 g/dL); active major bleeding or severe coagulation disorders precluding blood withdrawal; uncompensated cardiovascular disease, including unstable angina, recent myocardial infarction, severe heart failure or uncontrolled arrhythmias; severe renal or hepatic failure; active malignant disease requiring immediate chemotherapy or radiotherapy; acute systemic infection requiring emergency hospitalization or intensive care; severe autoimmune disease during an active flare; chronic immunosuppressive treatment judged by the investigator to interfere with study procedures; inability to obtain adequate peripheral venous access; known hypersensitivity to heparin or history of heparin-induced thrombocytopenia; participation in another interventional clinical trial within the previous three months; inability to comply with the treatment schedule or follow-up visits; refusal or withdrawal of informed consent at any stage of the study. Patients who discontinued oxygen–ozone treatment before completing the scheduled eight-session protocol, missed more than two treatment sessions, or were lost to follow-up before completion of the predefined outcome assessment were considered dropouts and were excluded from the per-protocol analysis.

### 4.5. Primary and Secondary Endpoints

The primary endpoint was the proportion of patients achieving clinical treatment success at the end of the four-week treatment period. Clinical success was defined as the simultaneous achievement of: (a) ≥50% reduction in the presence of the MDR bacteria; (b) significant reduction or complete resolution of the principal signs and symptoms of inflammation (pain, swelling, erythema, local warmth, or purulent discharge, according to the underlying pathology); (c) absence of clinical evidence of disease progression or requirement for escalation of antibiotic therapy; and (d) no indication for unplanned surgical intervention related to the treated condition.

Secondary endpoints included: (a) changes in inflammatory biomarkers, mainly including C-reactive protein (CRP) and erythrocyte sedimentation rate (ESR); (b) microbiological response, defined as eradication or reduction in the causative pathogen on follow-up cultures when clinically indicated; (c) reduction in the duration or cumulative dose of systemic antibiotic therapy; (d) recurrence of infection during the six-month follow-up period; (e) quality-of-life improvement assessed using validated patient-reported outcome measures appropriate for the underlying disease; (f) incidence of hospitalization or surgical intervention after initiation of treatment and life expectancy; (g) safety and tolerability of oxygen–ozone therapy, including the incidence of adverse events and serious adverse events; and (h) in the subgroup of patients with chronic prosthetic joint infection, qualitative and quantitative assessment of bacterial biofilm disruption, evaluated by microbiological analysis together with scanning electron microscopy and confocal laser scanning microscopy of retrieved prosthetic specimens or representative biofilm samples, when available.

### 4.6. Microbiological Processing and Confirmation of Bacterial Isolates

Clinical specimens were analysed in the diagnostic microbiology laboratories of public hospitals located in the Bergamo, Brescia, and Milan districts following the respective institutional standard operating procedures. For the primary isolation or presumptive identification of the microorganisms of interest, samples were inoculated onto selective culture media appropriate for each pathogen. Specifically, CHROMagar™ MRSA (CHROMagar, Paris, France) was used for the detection of methicillin-resistant *Staphylococcus aureus* (MRSA), cetrimide agar (BioMérieux, Marcy l’Étoile, France) for *Pseudomonas aeruginosa*, CHROMID^®^ CARBA agar (BioMérieux, Marcy l’Étoile, France) for carbapenem-resistant *Klebsiella pneumoniae*, and CHROMID^®^ ESBL agar (BioMérieux, Marcy l’Étoile, France) for the presumptive recovery of extended-spectrum β-lactamase (ESBL)-producing Enterobacterales. Culture media were incubated under routine laboratory conditions in accordance with the manufacturers’ recommendations and the standard protocols adopted by each participating laboratory.

After incubation, culture plates were evaluated for phenotypic characteristics consistent with the expected organisms, including colony morphology, pigment formation, and chromogenic reactions when applicable. Mauve to pink colonies growing on CHROMagar™ MRSA were regarded as presumptive MRSA isolates, whereas typical pigmented colonies developing on cetrimide agar were considered compatible with *P. aeruginosa*. Growth on CHROMID^®^ CARBA agar was interpreted as suggestive of carbapenem-resistant Enterobacterales, including *K. pneumoniae*. Whenever indicated, presumptive colonies were sub-cultured to obtain pure isolates before undergoing confirmatory identification using validated routine microbiological procedures employed in the participating diagnostic laboratories.

Species-level identification was performed in the accredited microbiology laboratories participating in the study using the routine diagnostic platforms available at each center, including matrix-assisted laser desorption/ionization time-of-flight mass spectrometry (MALDI-TOF MS), where available, and other validated automated microbiological identification systems. All laboratories followed their institutional standard operating procedures and applicable EUCAST/CLSI recommendations before antimicrobial susceptibility testing.

Bacterial burden was quantified as colony-forming units per milliliter (CFU/mL) using standard quantitative culture techniques. Clinical specimens obtained from the site of infection were serially diluted in sterile physiological saline and plated onto appropriate microbiological media according to the pathogen under investigation. Following incubation under standard laboratory conditions, visible colonies were counted on plates containing 30–300 colonies, and bacterial concentrations were calculated as CFU/mL by multiplying the colony count by the corresponding dilution factor. Quantitative cultures were performed at baseline and at each scheduled follow-up visit using the same microbiological procedures. Longitudinal changes in bacterial burden were evaluated using absolute CFU/mL values and log_10_(CFU/mL + 1)-transformed data to accommodate skewed distributions and zero counts. Percentage reduction was calculated relative to the baseline bacterial load, whereas microbiological clearance was defined as the absence of detectable bacterial growth on quantitative culture. All quantitative microbiological analyses were performed using the same methodology throughout the study to ensure consistency between follow-up assessments.

Confirmation of antimicrobial resistance was subsequently performed using standard antimicrobial susceptibility testing and, where routinely implemented, established assays for resistance marker detection. Methicillin resistance in *S. aureus* and carbapenem resistance in *K. pneumoniae* were verified according to the diagnostic workflow of each laboratory. The definitive microbiological report was therefore based on confirmatory organism identification together with antimicrobial susceptibility or resistance testing, rather than on colony appearance or growth characteristics observed on selective or chromogenic media alone.

### 4.7. Antibiogram and Antimicrobial Susceptibility Testing

Antimicrobial susceptibility testing (AST) was performed on bacterial isolates recovered during clinical follow-up using standardized microbiological procedures. Isolates were identified at the species level, and susceptibility testing was carried out according to internationally accepted clinical breakpoints (e.g., EUCAST or CLSI, depending on the laboratory protocol). Minimum inhibitory concentrations (MICs) were determined for a panel of clinically relevant antimicrobial agents, and isolates were classified as susceptible (S), intermediate (I), or resistant (R) according to the corresponding interpretive criteria. For the present analysis, antibiograms obtained at one and two years of follow-up were combined. The resistance proportion for each antibiotic was calculated as the number of resistant (R) isolates divided by the total number of isolates tested for that antibiotic, while susceptible and intermediate isolates were considered non-resistant. Antibiotics were subsequently ranked according to their overall resistance proportion and visualized as a horizontal bar chart.

Quality assurance for antimicrobial susceptibility testing was performed using the reference quality-control strains routinely employed by the participating diagnostic microbiology laboratories in accordance with EUCAST/CLSI recommendations. These included *Staphylococcus aureus* ATCC 29213 (and/or ATCC 25923 for disk diffusion), *Escherichia coli* ATCC 25922, and *Pseudomonas aeruginosa* ATCC 27853, where appropriate. The observed quality-control results fell within the accepted interpretive ranges specified by the corresponding guideline.

For the purposes of this study, multidrug-resistant (MDR) bacteria were defined according to the international consensus definition of Magiorakos et al. as isolates demonstrating non-susceptibility to at least one agent in three or more antimicrobial categories [44].

### 4.8. Major Inflammatory Markers

Systemic inflammation was monitored by measuring erythrocyte sedimentation rate (ESR) and C-reactive protein (CRP) at baseline and during the scheduled follow-up visits. ESR was determined using an automated Westergren-based method (TEST1 2.0 System, Alifax, Padova, Italy), while CRP concentrations were measured by automated immunoturbidimetric assay (Beckman Coulter AU/DxC AU analyzers, Brea, CA, USA), according to the manufacturers’ instructions and routine laboratory procedures. Both biomarkers were analyzed in the accredited clinical laboratories participating in the study and were used to monitor the longitudinal evolution of systemic inflammatory activity during treatment.

### 4.9. Oxygen-Ozone Major Autohaemotherapy (SIOOT^®^-O_2_-O_3_-MAHT)

Patients included in the present study had been discharged from hospital but continued to experience recurrent or persistent multidrug-resistant bacterial infections despite prolonged guideline-directed antibiotic therapy, often requiring repeated modifications of the antimicrobial regimen over a period of at least two years. Patients voluntarily sought adjunctive oxygen–ozone therapy after receiving information from their treating physicians or from publicly available scientific and medical sources. Consequently, the present investigation was designed as a prospective longitudinal pre–post intervention study in which each patient served as his or her own historical control rather than as a randomized controlled trial. Medical ozone was generated immediately before administration using a certified medical ozone generator (Multiossigen S.p.A., Gorle, Bergamo, Italy) equipped with an integrated ultraviolet spectrophotometric monitoring system operating at 254 nm to continuously verify the ozone concentration produced from medical-grade oxygen. For each treatment session, 200 mL of autologous venous blood was collected into a sterile, sodium-citrate, phthalate-free, ozone-resistant closed collection system (SANO3). The blood was then exposed ex vivo to an equal volume of an oxygen–ozone gas mixture containing ozone at concentrations ranging from 30 to 40 μg/mL, in accordance with the standardized protocols of the Italian Scientific Society of Oxygen–Ozone Therapy (SIOOT^®^) [40,41]. This concentration range was selected because it lies within the therapeutic window commonly adopted for systemic major autohemotherapy, providing sufficient biological stimulation while minimizing excessive oxidative stress. Within this predefined range, the exact ozone concentration was selected according to the patient’s clinical condition, type and severity of infection, inflammatory status, comorbidities, and individual tolerance to treatment, following the standardized SIOOT^®^ therapeutic recommendations for major autohemotherapy. After gentle mixing for approximately 5 min to ensure homogeneous gas dissolution and formation of secondary reactive oxygen species and lipid oxidation products, the ozonated blood was reinfused intravenously at a controlled infusion rate of approximately 80 drops/min. The induction treatment consisted of a minimum of eight and a maximum of twelve major autohemotherapy sessions administered twice weekly over four consecutive weeks while patients continued receiving guideline-directed antibiotic therapy without interruption. Thereafter, patients entered a maintenance phase with scheduled SIOOT^®^-O_2_-O_3_-MAHT sessions during the subsequent 11 months according to their clinical evolution and microbiological response. For statistical consistency, all longitudinal analyses were standardized to a common treatment schedule consisting of eight induction sessions followed by one maintenance session per month for a total follow-up period of one year.

The ozone concentration range and treatment schedule were selected according to published recommendations for systemic oxygen–ozone major autohemotherapy and the standardized clinical protocols of the Italian Scientific Society of Oxygen–Ozone Therapy (SIOOT^®^), which recommend concentrations between 30 and 40 μg/mL for chronic inflammatory and infectious disorders treated by major autohemotherapy.

The selection of ozone concentrations between 30 and 40 μg/mL for major autohemotherapy is supported by the high antioxidant buffering capacity of human blood [40,41,42]. Therapeutic ozone reacts immediately upon contact with blood and does not persist as free ozone because it is rapidly consumed by plasma antioxidants and cellular redox systems. Reduced glutathione, present at intracellular concentrations of approximately 200–625 μM, together with plasma uric acid (200–400 μM), ascorbate, albumin thiol groups, and other endogenous antioxidants, provides a buffering capacity that greatly exceeds the oxidative stimulus generated by ozone at therapeutic concentrations. Consequently, ozone administered within this concentration range does not induce uncontrolled oxidative damage but rather produces a transient and tightly regulated oxidative challenge, leading to the formation of low concentrations of hydrogen peroxide and lipid oxidation products that function as secondary signaling molecules. This controlled redox stimulus activates adaptive antioxidant pathways (hormesis), including the Nrf2 signaling cascade, while preserving cellular integrity. Consistent with this biological rationale, major autohemotherapy performed with ozone concentrations of 30–40 μg/mL according to standardized clinical protocols has demonstrated a favorable safety profile in numerous clinical studies, with a very low incidence of treatment-related adverse events.

### 4.10. Isolation and Culture of Peripheral Blood Monocyte-Derived Macrophages

Peripheral blood samples were collected one week after completion of the treatment protocol. Peripheral blood mononuclear cells (PBMCs) were isolated by Ficoll–Paque density-gradient centrifugation (GE Healthcare).

Monocytes were purified by plastic adherence and cultured in RPMI-1640 supplemented with: 10% heat-inactivated foetal bovine serum (FBS), 2 mM L-glutamine, 100 U/mL penicillin, 100 μg/mL streptomycin. During this phase cells were treated with PBS (control group) and with PBS containing 40 μg/mL O_3_ and incubated overnight at 5% CO_2_. Ozone was provided by BMT 803 BT-A from BMT Messtechnik, Germany, only for scientific and experimental purposes (not medical), which combines a bench-top corona-discharge ozone generator with a dedicated dual-beam UV ozone analyser.

Cells were differentiated into macrophages during seven days in the presence of recombinant human macrophage colony-stimulating factor (M-CSF, 50 ng/mL), with medium replacement every 48 h. Before infection, antibiotics were removed by washing twice with antibiotic-free RPMI.

### 4.11. Preparation of Fluorescent MRSA

A clinical multidrug-resistant methicillin-resistant *Staphylococcus aureus* (MRSA) isolate recovered from prosthetic joint infection was cultured overnight in tryptic soy broth at 37 °C.

Bacteria were labelled using the nucleic acid fluorescent probe SYTO™ 9 (Thermo Fisher Scientific) according to the manufacturer’s instructions, washed three times in sterile PBS, and adjusted spectrophotometrically to the desired bacterial concentration. Macrophages were infected at a multiplicity of infection (MOI) of 10 bacteria per macrophage and incubated for 60 min at 37 °C under 5% CO_2_.

Following incubation, extracellular bacteria were removed by repeated washing with PBS. Where indicated, extracellular fluorescence was quenched with trypan blue (0.2%) immediately before image acquisition to distinguish adherent from internalized bacteria.

### 4.12. Immunofluorescent Staining

Macrophages were fixed with 4% paraformaldehyde for 15 min at room temperature. Following permeabilization using 0.1% Triton X-100, nonspecific binding was blocked with 3% bovine serum albumin (BSA). Macrophages were stained using: (a) mouse anti-human CD68 monoclonal antibody, (b) Alexa Fluor^®^ 488-conjugated secondary antibody. Cell nuclei were counterstained using DAPI (4′,6-diamidino-2-phenylindole). Slides were mounted using ProLong™ Gold Antifade Mountant.

### 4.13. Confocal Laser Scanning Microscopy

Fluorescence images were acquired using a confocal laser scanning microscope (insert manufacturer and model; e.g., Leica SP8, Zeiss LSM980, or Nikon A1R) (Leica SP8: Leica Microsystems CMS GmbH, Wetzlar, Germany. Zeiss LSM 980: Carl Zeiss Microscopy GmbH, Jena, Germany. Nikon A1R: Nikon Corporation, Tokyo, Japan) equipped with a 63× oil immersion objective (NA 1.40). All images were collected using identical acquisition settings for every experimental group. Fluorophores were excited at 405 nm for DAPI and 488 nm for Alexa Fluor 488 and SYTO™ 9, and sequential image acquisition was used throughout to eliminate fluorescence bleed-through. For each experimental condition, at least 15 randomly selected microscopic fields from three independent experiments were analyzed. Z-stack images were acquired at 0.3–0.5 μm optical intervals throughout the entire macrophage thickness, and three-dimensional reconstructions together with orthogonal XZ and YZ projections were generated using LAS X, ZEN, or FIJI/ImageJ software v2.16.0/ImageJ 1.54p. to verify intracellular bacterial localization.

Phagocytosis was quantified from confocal images by investigators blinded to treatment allocation. For each sample, at least 200 macrophages were evaluated, and the percentage of phagocytic macrophages, defined as (macrophages containing ≥1 bacterium/total macrophages) × 100, the mean number of intracellular bacteria per macrophage, and the phagocytic index, calculated as (macrophages containing intracellular bacteria/total macrophages) × mean intracellular bacteria per macrophage, were determined. Only bacteria completely enclosed within the CD68-positive macrophage cytoplasm in orthogonal confocal sections were considered internalized.

To verify that intracellular bacteria resided within mature phago-lysosomal compartments rather than remaining adherent to the cell surface, macrophages were immunostained with anti-human lysosomal-associated membrane protein-1 (LAMP1; clone H4A3, Thermo Fisher Scientific) or, in selected experiments, anti-Rab7 monoclonal antibody (Cell Signaling Technology), followed by Alexa Fluor^®^ 647-conjugated secondary antibodies. Colocalization between intracellular MRSA and LAMP1- or Rab7-positive compartments was assessed by sequential confocal imaging, while orthogonal (XZ and YZ) projections and three-dimensional reconstructions confirmed complete intracellular localization within phago-lysosomal compartments. Functional phagocytosis was further evaluated using MRSA labeled with the pH-sensitive fluorescent probe pHrodo™ Red STP Ester (Thermo Fisher Scientific) according to the manufacturer’s instructions. Unlike conventional nucleic acid stains such as SYTO™ 9, pHrodo™ exhibits minimal fluorescence at neutral extracellular pH but becomes progressively fluorescent after phagosome acidification, thereby serving as a selective indicator of bacterial internalization into mature acidic phagolysosomes. pHrodo fluorescence intensity was quantified under identical acquisition settings for all experimental groups.

Viable intracellular bacteria were quantified using a gentamicin protection assay. Following 60 min of macrophage–bacteria interaction, extracellular bacteria were removed by extensive washing with phosphate-buffered saline (PBS), and the cultures were incubated with gentamicin (100 μg/mL for 60 min) to eliminate remaining extracellular MRSA. Cells were then washed three times with PBS, lysed with 0.1% Triton X-100, and serial dilutions of the lysates were plated onto tryptic soy agar and incubated overnight at 37 °C. Colony-forming units (CFU) were enumerated and expressed as intracellular viable MRSA per 10^6^ macrophages, providing a functional measure of bacterial uptake and intracellular survival following antibiotic treatment alone or in combination with oxygen–ozone therapy.

Confocal images were analyzed using an automated image-analysis pipeline developed in FIJI/ImageJ (National Institutes of Health, Bethesda, MD, USA), with selected analyses confirmed using CellProfiler (Broad Institute, Cambridge, MA, USA) and Imaris (Oxford Instruments, Zurich, Switzerland). Macrophages were segmented using the CD68 fluorescence channel, whereas bacterial objects were identified from the SYTO™ 9 or pHrodo™ fluorescence channel by intensity thresholding and watershed segmentation. Only bacterial signals completely enclosed within macrophage cytoplasmic boundaries in reconstructed three-dimensional image stacks were classified as internalized. Automated image analysis generated the percentage of bacteria-containing macrophages, total intracellular bacterial fluorescence per macrophage, mean intracellular bacteria per macrophage, phagocytic index, percentage of MRSA colocalizing with LAMP1- or Rab7-positive compartments, and pHrodo fluorescence intensity as an indicator of phagosome acidification. Identical threshold settings were applied to all experimental groups, and investigators responsible for image processing and quantitative analyses remained blinded to treatment allocation to minimize observer bias and ensure reproducibility.

### 4.14. Scanning Electron Microscopy (SEM) Analysis of MRSA Biofilms on Titanium Surfaces

Titanium alloy discs were used as prosthetic-surface models and sterilized before bacterial inoculation. A clinical multidrug-resistant MRSA isolate was cultured overnight in tryptic soy broth at 37 °C, adjusted to approximately 10^7^ CFU/mL, and seeded onto the titanium discs. Biofilms were allowed to mature for 24–48 h at 37 °C under static conditions.

After biofilm maturation, samples were divided into two groups: untreated MRSA biofilm controls and ozone-treated biofilms. In the ozone-treated group, discs were exposed to a freshly generated medical oxygen–ozone mixture at 40 μg/mL for the predefined exposure time under controlled conditions. Control discs were handled identically but without ozone exposure.

Following treatment, discs were gently washed with sterile phosphate-buffered saline to remove non-adherent bacteria. Biofilms were fixed with 2.5% glutaraldehyde in 0.1 M cacodylate buffer for 2 h at 4 °C, rinsed three times in buffer, and post-fixed with 1% osmium tetroxide for 1 h. Samples were then dehydrated through graded ethanol concentrations, dried by critical-point drying, mounted on aluminum stubs, and sputter-coated with gold/palladium.

Scanning electron microscopy was performed using a field-emission SEM operated at 5–10 kV. Images were acquired at comparable magnifications under identical acquisition settings. Representative micrographs were obtained from randomly selected areas of each titanium disc.

Biofilm morphology was assessed qualitatively by evaluating bacterial clustering, extracellular polymeric substance density, surface coverage, matrix disruption, exposed titanium topography, and cellular debris. Untreated samples showed dense MRSA biofilm coverage with abundant extracellular polymeric substance and tightly packed coccoid bacterial clusters. In contrast, oxygen–ozone-treated samples showed reduced biofilm biomass, disrupted extracellular matrix, exposed titanium surface, scattered bacterial aggregates, and debris compatible with ozone-induced oxidative biofilm disruption.

### 4.15. Statistics

Statistical analyses were performed on 257 patients who completed all scheduled microbiological follow-up assessments. Continuous variables were examined for distributional characteristics using graphical inspection (histograms and Q–Q plots) together with the Shapiro–Wilk normality test. Because colony-forming unit (CFU/mL) counts exhibited marked right-skewness and included zero values during follow-up, microbiological data were logarithmically transformed as log_10_(CFU/mL + 1) before inferential analyses to stabilize the variance and accommodate zero counts.

Descriptive statistics are presented as arithmetic mean ± standard deviation (SD), median with interquartile range (IQR), minimum–maximum range, and geometric mean with 95% confidence intervals (95% CI). Geometric means were calculated by averaging log_10_(CFU/mL + 1) values followed by back-transformation to the original measurement scale. The proportion of positive cultures at each follow-up visit was also calculated. Changes in bacterial load over time were evaluated using the Friedman test for repeated measures, an appropriate non-parametric alternative to repeated-measures ANOVA for paired observations that do not satisfy normality assumptions.

Three analyses were performed: (a) the early treatment phase (baseline, 1 week and 1 month), (b) the long-term follow-up phase (1, 2, 3, 6 and 12 months), and (c) the complete longitudinal follow-up from baseline to 12 months.

Effect sizes for Friedman analyses were quantified using Kendall’s coefficient of concordance (Kendall’s W), interpreted according to conventional thresholds (0.1 small, 0.3 moderate, and ≥0.5 large effect). When significant overall differences were identified, pairwise comparisons between time points were performed using the Wilcoxon signed-rank test for paired samples on log-transformed CFU values.

To control the family-wise type I error rate arising from multiple comparisons, *p* values were adjusted using the Holm sequential correction procedure. Treatment effects were additionally quantified by calculating Cohen’s *d_z_* for paired observations and the standardized Wilcoxon effect size (*r*), thereby providing estimates of both parametric and rank-based effect magnitude. Percentage reduction in bacterial burden and geometric fold reduction were calculated relative to both the pre-treatment baseline and the 1-month post-treatment reference point. Percentage reduction was calculated as:

$$Reduction\;\%=(1-\frac{{CFU}_{follow\;up}}{{CFU}_{reference}})\times 100$$

whereas geometric fold reduction was obtained from the ratio of geometric means between the reference and follow-up measurements.

Longitudinal bacterial eradication was further described by calculating the proportion of microbiologically positive cultures at each follow-up visit. Because every patient completed the planned observation schedule, no missing data imputation or censoring procedures were required.

All statistical tests were two-sided, and statistical significance was defined as *p* < 0.05. Analyses were performed using R version 4.4.0 (R Foundation for Statistical Computing, Vienna, Austria) with the packages rstatix, survival, survminer, and ggplot2 (along with IBM SPSS Statistics version 29.0). Data used for statistics can be found as Table S1.

### 4.16. Hypothetical Survival Forecasting

To illustrate the potential clinical implications of improved microbiological control, a hypothetical survival forecast was constructed using a Kaplan–Meier-style graphical representation. This model was not derived from patient-level survival data collected during the present study and therefore should not be interpreted as an actual survival analysis. Instead, it represents a conceptual projection designed to visualize how differences in infection control might theoretically influence short-term survival. An initial hypothetical cohort of 100 patients per treatment group was assumed. Predetermined cumulative survival probabilities were assigned at 0, 7, 14, 28, 60, and 90 days, corresponding to projected survival of 100%, 85%, 70%, 55%, 45%, and 35% for the antibiotic-only group and 100%, 98%, 90%, 85%, 80%, and 75% for the adjunctive ozone group. These projected values were displayed using the conventional stepwise Kaplan–Meier graphical format, in which survival remains constant between successive observation times.

The projected cumulative survival function, (S(t)), was expressed according to the Kaplan–Meier product-limit estimator,

$$\hat{S}(t)=\prod_{t_{i}⩽t}(1-\frac{d_{i}}{n_{i}})$$

where (n_i_) denotes the number of individuals hypothetically remaining at risk immediately before time (t_i_), and (d_i_) the projected number of deaths occurring during the corresponding interval. In the present illustration, however, the values of (d_i_) and (n_i_) were not obtained from observed event-time data, but were back-calculated from predefined cumulative survival percentages solely for graphical visualization.

No censoring, log-rank testing, Cox proportional hazards regression, hazard ratios, confidence intervals, or median survival estimates were calculated because individual patient-level survival data were unavailable. Consequently, the figure should be regarded exclusively as a conceptual survival forecast illustrating a hypothetical clinical scenario rather than as evidence of an observed survival benefit attributable to adjunctive oxygen–ozone therapy. Confirmation of any survival advantage will require prospective randomized clinical trials with appropriately collected time-to-event data.

## 5. Conclusions

This pilot study demonstrates that adjunctive oxygen–ozone major autohemotherapy (SIOOT^®^-O_2_-O_3_-MAHT), administered according to standardized SIOOT protocols alongside conventional antibiotic therapy, was associated with a rapid, profound, and durable improvement in patients with chronic multidrug-resistant bacterial infections. Treatment produced a marked reduction in bacterial burden, culminating in complete microbiological clearance at one year, while simultaneously promoting progressive normalization of the inflammatory biomarkers ESR and CRP. Mechanistic investigations further showed that ozone enhanced macrophage phagocytosis, phagolysosomal maturation, intracellular bacterial killing, and disruption of mature MRSA biofilms, providing biologically plausible explanations for the observed clinical and microbiological outcomes. These findings support the concept that ozone acts through complementary mechanisms that include direct oxidative antimicrobial activity, biofilm degradation, modulation of host redox signaling, and reinforcement of innate immune defenses, thereby enhancing the effectiveness of conventional antibiotics.

Although the present study provides encouraging evidence for the potential role of oxygen–ozone therapy as an adjunctive treatment, its findings should be interpreted within the limitations of a non-randomized pilot investigation. Confirmation of efficacy and safety will require well-designed multicenter randomized controlled trials incorporating standardized treatment protocols, patient-level survival analyses, microbiological and immunological endpoints, long-term follow-up, and cost-effectiveness assessments. If validated in larger controlled studies, adjunctive oxygen–ozone therapy may represent a valuable complementary strategy for managing chronic infections caused by multidrug-resistant pathogens, particularly in patients with persistent, recurrent, or biofilm-associated infections for whom conventional antibiotic therapy alone has proven insufficient.

## Figures and Tables

**Table 1:** Distribution of major pathologies and MDR micro-organisms in the recruited patients.

**(A)**		
**Pathology Category**	**n**	**%**
Chronic/recurrent urinary tract infections	54	21.0%
Chronic skin/soft-tissue infections, ulcers, wound infections	46	17.9%
Chronic sinusitis/ENT infections	34	13.2%
Chronic bronchitis/recurrent respiratory infections	31	12.1%
Dental/periodontal/maxillofacial infections	29	11.3%
Prosthetic/orthopedic/osteoarticular infections	24	9.3%
Chronic gastrointestinal/biliary inflammatory-infectious conditions	18	7.0%
Gynecological/pelvic recurrent infections	13	5.1%
Other chronic inflammatory-infectious conditions	8	3.1%
Total	257	100%
**(B)**		
**Multi-Drug-Resistant (MDR)** **microorganisms**	**n**	**%**
MRSA (methicillin-resistant *Staphylococcus aureus*)	77	30.0%
Extended-spectrum β-lactamase-producing *Escherichia coli* (ESBL-producing *Escherichia coli*)	46	17.9%
MDR *Pseudomonas aeruginosa*	39	15.2%
Extended-spectrum β-lactamase-producing/MDR *Klebsiella pneumoniae*	36	14.0%
MDR *Enterococcus faecalis/faecium*	24	9.3%
Coagulase-negative staphylococci (biofilm-forming)	26	10.1%
MDR *Proteus mirabilis*/other Enterobacterales	2	0.8%
MDR anaerobic or mixed polymicrobial flora	4	1.5%
MDR *Acinetobacter baumannii* or other non-fermenters	2	0.8%
*Candida albicans*	1	0.4%
Total	257	100%

**Table 2:** Longitudinal descriptive statistics.

Time Point	n	Geometric Mean (95% CI), CFU/mL	Mean ± SD, CFU/mL	Median (IQR), CFU/mL	Range, CFU/mL	Positive Cultures, n (%)
**Before**	257	6.43 × 10^6^ (6.02 × 10^6^–6.87 × 10^6^	7.80 × 10^6^ ± 8.53 × 10^6^	6.37 × 10^6^ (5.08 × 10^6^–7.86 × 10^6^)	1 × 10^5^–8 × 10^7^	257 (100.0)
**1 week**	257	24,934 (20,554–30,249)	1.83 × 10^5^ ± 8.03 × 10^5^	37,500 (6450–69,400)	3280–6.50 × 10^6^	257 (100.0)
**1 month**	257	1793 (1561–2059)	3687 ± 6114	1487 (1042–3142)	140–44,000	257 (100.0)
**2 months**	257	427 (384–475)	655 ± 892	430 (250–568)	45–10,000	257 (100.0)
**3 months**	257	37.7 (28.4–49.8)	152 ± 265	100 (20–145)	0–3000	195 (75.9)
**6 months**	257	0.95 (0.60–1.37)	15.7 ± 48.1	0 (0–0)	0–450	39 (15.2)
**1 year**	257	0	0 ± 0	0 (0–0)	0–0	0 (0.0)

CFU/mL distributions are summarized using both arithmetic and log-based geometric statistics. Geometric means were estimated from log_10_(CFU/mL + 1) and back-transformed.

**Table 3:** Reduction from 1-month reference value.

Comparison	n	Mean log_10_ Reduction	Median log_10_ Reduction	Mean Reduction (%)	Median Reduction (%)	Geometric Fold Reduction	Wilcoxon W	Cohen dz	Effect Size r	Holm-Adjusted *p*
**1 month vs. 2 months**	257	0.622	0.591	71.30	74.41	4.2×	84	2.20	0.86	**<0.0001**
**1 month vs. 3 months**	257	1.666	1.412	94.88	96.22	46.4×	1	2.31	0.87	**<0.0001**
**1 month vs. 6 months**	257	2.965	3.101	99.77	100.00	922.2×	0	4.91	0.87	**<0.0001**
**1 month vs. 1 year**	257	3.254	3.173	100.00	100.00	1793.7×	0	6.66	0.87	**<0.0001**

Positive log_10_ reduction indicates lower CFU/mL than the 1-month reference value. *p* values are from paired Wilcoxon signed-rank tests on log_10_(CFU/mL + 1), with Holm correction.

**Table 4:** Overall repeated-measures tests.

Analysis Set	Test	Statistic (χ^2^)	df	*p* Value	Kendall W
**Initial treatment phase: Before, 1 week, 1 month**	Friedman	506.125	2	**<0.0001**	0.985
**Long-term phase: 1, 2, 3, 6 and 12 months**	Friedman	986.527	4	**<0.0001**	0.960
**Complete follow-up: Before to 1 year**	Friedman	1519.180	6	**<0.0001**	0.985

Kendall W is reported as the repeated-measures effect size for the Friedman test.

**Table 5:** Reduction from pre-treatment baseline.

Comparison	n	Mean log_10_ Reduction	Median log_10_ Reduction	Mean Reduction (%)	Median Reduction (%)	Geometric Fold Reduction	Wilcoxon W	Cohen dz	Effect Size r	Holm-Adjusted *p*
**Before vs. 1 week**	257	2.411	2.252	98.53	99.44	257.9×	0	3.77	0.87	**<0.0001**
**Before vs. 1 month**	257	3.555	3.597	99.95	99.97	3585.4×	0	7.36	0.87	**<0.0001**
**Before vs. 2 months**	257	4.176	4.199	99.99	99.99	15,012.3×	0	9.68	0.87	**<0.0001**
**Before vs. 3 months**	257	5.221	4.886	100.00	100.00	166,264.7×	0	5.33	0.87	**<0.0001**
**Before vs. 6 months**	257	6.519	6.769	100.00	100.00	3,306,398.1×	0	9.06	0.87	**<0.0001**
**Before vs. 1 year**	257	6.808	6.804	100.00	100.00	6,431,071.2×	0	28.87	0.87	**<0.0001**

**Statistical notes.** CFU/mL values were analyzed after log_10_(CFU/mL + 1) transformation to accommodate skewness and zero counts. Descriptive data are shown as geometric mean (95% CI), arithmetic mean ± SD, median (IQR), and range. Pairwise comparisons used Wilcoxon signed-rank tests with Holm adjustment. Cohen dz is reported for paired log_10_ differences; r is an approximate standardized Wilcoxon effect size derived from the two-sided *p* value.

## Data Availability

Data are available on request.

## Supplementary Materials

The following supporting information can be downloaded at: https://www.mdpi.com/article/10.3390/antibiotics15080768/s1, Figure S1: Figure 6 original photos; File S1: Informed_Consent_for_Participation; File S2: Consent_for_Publication; Table S1: Data used for statistics (DATI GLOBALI).

## Abbreviations

The following abbreviations are used in this manuscript: AMR, antimicrobial resistance; ARE, antioxidant response element; BSA, bovine serum albumin; CFU, colony-forming unit; CI, confidence interval; CLSI, Clinical and Laboratory Standards Institute; CRP, C-reactive protein; CV, coefficient of variation; DAPI, 4′,6-diamidino-2-phenylindole; DNA, deoxyribonucleic acid; EPS, extracellular polymeric substance; ESBL, extended-spectrum β-lactamase; ESR, erythrocyte sedimentation rate; FBS, fetal bovine serum; FIJI, Fiji Is Just ImageJ; ICSH, International Council for Standardization in Haematology; IQR, interquartile range; IRB, Institutional Review Board; Keap1, Kelch-like ECH-associated protein 1; LAMP1, lysosome-associated membrane protein 1; MAHT, major autohemotherapy; MARCO, macrophage receptor with collagenous structure; M-CSF, macrophage colony-stimulating factor; MDR, multidrug-resistant; MOI, multiplicity of infection; MRSA, methicillin-resistant *Staphylococcus aureus*; NA, not available; NF-κB, nuclear factor kappa B; Nrf2, nuclear factor erythroid 2-related factor 2; NSAIDs, non-steroidal anti-inflammatory drugs; SIOOT^®^-O_2_-O_3_-MAHT, oxygen–ozone major autohemotherapy according SIOOT^®^; PBS, phosphate-buffered saline; PBMCs, peripheral blood mononuclear cells; PI, phagocytic index; Rab7, Ras-related protein Rab-7; ROS, reactive oxygen species; SD, standard deviation; SEM, scanning electron microscopy; SIOOT, Italian Scientific Society of Oxygen-Ozone Therapy (Società Scientifica Italiana di Ossigeno-Ozonoterapia); SoxRS, superoxide response regulon; 4-HNE, 4-hydroxynonenal.
