# Supplementary material for: SIOOT® Adjunct Oxygen-Ozone Therapy Against Multidrug-Resistant Bacteria: A Pilot Study of 257 Cases
Source: Antibiotics (Basel). 2026 Aug 10;15(8):768. doi: 10.3390/antibiotics15080768 (PMC13509617; doi:10.3390/antibiotics15080768)
Supplement: Supplementary file 1 [file antibiotics-15-00768-s001.zip › antibiotics-4472566-supplementary/File S1 SIOOT_Informed_Consent_for_Participation.pdf]

Italian Scientific Society of Oxygen–Ozone Therapy (SIOOT)
Informed Consent for Participation in a Research Study

Study Title: ______________________________________

Principal Investigator: ______________________________

Institution: ______________________________________

I confirm that I have received adequate information regarding the purpose of the study, its procedures, expected duration, potential benefits, foreseeable risks and discomforts, confidentiality of my personal data, and my right to withdraw at any time without any consequences for my medical care.

I understand that participation is voluntary. I have had the opportunity to ask questions, and all my questions have been answered satisfactorily.

I consent to the collection and processing of my data for scientific research in accordance with applicable data protection regulations (including GDPR where applicable).

Participant Name: ___________________________________

Date of Birth: ___________________________________

Study ID (optional): ___________________________________

Signature of Participant: ___________________________________

Date: ___________________________________

Investigator/Witness Signature: ___________________________________
