# Supplementary material for: SIOOT® Adjunct Oxygen-Ozone Therapy Against Multidrug-Resistant Bacteria: A Pilot Study of 257 Cases
Source: Antibiotics (Basel). 2026 Aug 10;15(8):768. doi: 10.3390/antibiotics15080768 (PMC13509617; doi:10.3390/antibiotics15080768)
Supplement: Supplementary file 1 [file antibiotics-15-00768-s001.zip › antibiotics-4472566-supplementary/File S2 SIOOT_Consent_for_Publication.pdf]

# Italian Scientific Society of Oxygen–Ozone Therapy (SIOOT)
# Consent for Publication

Study/Manuscript Title: ________________________________

I authorize the publication of my anonymized clinical information, laboratory results, medical images, photographs (where specifically indicated), and other research data for scientific and educational purposes.

I understand that:
• My identity will not be intentionally disclosed.
• Complete anonymity cannot be absolutely guaranteed.
• I may withdraw this consent before publication by notifying the investigators in writing.
• This consent applies to publication in scientific journals, books, conference proceedings, websites, and educational materials.

Please tick as appropriate:

☐I consent to publication of anonymized clinical data.

☐I consent to publication of anonymized medical images.

☐I consent to publication of anonymized photographs (if applicable).

Participant Name: ________________________________

Signature of Participant: ________________________________

Date: ________________________________

Investigator/Witness Signature: ________________________________
